# In Situ Defect Healing Suppresses Mn Dissolution Chain Reactions in Aqueous Sodium-Ion Cathodes

**DOI:** 10.1007/s40820-026-02271-z

**Published:** 2026-06-30

**Authors:** Wenqing Du, Lin Xu, Yi Yang, Jingying Sun, Gongzheng Yang, Chengxin Wang

**Affiliations:** 1 State Key Laboratory of Optoelectronic Materials and Technologies, School of Materials Science and Engineering, Sun Yat-sen (Zhongshan) University, Guangzhou, 510275 People’s Republic of China; 2 Instrumental Analysis and Research Center, Sun Yat-sen (Zhongshan) University, Guangzhou, 510275 People’s Republic of China

**Keywords:** Aqueous sodium-ion batteries, Prussian blue analogs, Mn dissolution, Chain reactions, In situ defect healing

## Abstract

Sodium manganese hexacyanoferrate (Mn-HCF) is a promising cathode for aqueous sodium-ion batteries (ASIBs) due to its low cost and high theoretical capacity. However, its practical application is hindered by rapid capacity fading, which originates from Mn dissolution and the uneven lattice expansion induced by Jahn–Teller distortion and successive phase transitions. While strategies such as lattice doping, surface coating, and electrolyte additives have been explored to mitigate Mn dissolution, they merely delay rather than prevent the process. Moreover, the subsequent degradation reactions remain poorly understood. Herein, we elucidate a degradation chain reaction initiated by Mn dissolution. Dissolved Mn^2+^ ions catalyze interfacial water oxidation, generating protons that protonate the C≡N ligands of Fe(CN)_6_^4−/3−^. The subsequent ligand dissociation releases Fe^2+^/^3+^, which then react with residual Fe(CN)_6_^4−^ and Na^+^ to precipitate Na_x_Fe[Fe(CN)_6_] (Fe-HCF) on the electrode surface, ultimately leading to the lattice collapse of Mn-HCF. As this chain reaction continues, conventional approaches that only slow Mn dissolution are insufficient, and thus, the vacancies must be refilled in real time to halt the process. Accordingly, we introduce iron(III) trifluoromethanesulfonate (Fe(OTf)_3_) into a concentrated 17.6 m NaClO_4_ aqueous electrolyte. The Fe^3+^ ions rapidly occupy Mn vacancies as they form, thereby blocking the chain reaction at its source. A full cell incorporating the stabilized Mn-HCF cathode and a PTCDI (3,4,9,10-perylenetetracarboxylicdiimide) anode retains 80% of its initial capacity after 20,000 cycles at 2 A g^−1^, corresponding to an ultra-low-capacity fade rate of 0.001% per cycle that outperforms most reported ASIB cathodes.

**Supplementary Information:**

The online version contains supplementary material available at 10.1007/s40820-026-02271-z.

## Highlights

- A degradation chain reaction triggered by Mn dissolution is revealed, clarifying the continuous performance deterioration caused by Mn dissolution.
- A strategy for in situ surface repair is proposed, which achieves long-term stability through the synergistic action of high-concentration electrolyte and Fe^3+^ filling of Mn vacancies.
- After 20,000 cycles at a current density of 2 A g^−1^, the repaired electrode exhibited a capacity retention rate of 80%, outperforming most previously reported manganese-based aqueous sodium-ion battery cathodes.

**Supplementary Information:**

The online version contains supplementary material available at 10.1007/s40820-026-02271-z.

## Graphical abstract

**Figure:**
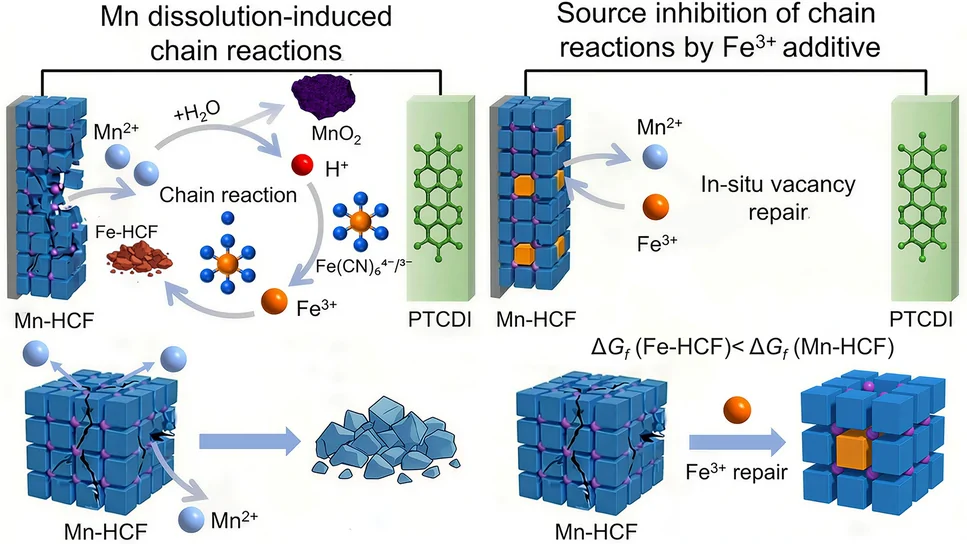

## Introduction

Aqueous sodium-ion batteries (ASIBs) offer superior safety, low cost, high ionic conductivity, and environmental friendliness, yet their energy density is fundamentally limited by the narrow electrochemical stability window of water (≈ 1.23 V) [1–4]. A viable route to raising the energy density while remaining within the aqueous stability window is to maximize the specific capacity and working potential of the cathode. Prussian blue analogs (PBAs, general formula A_*x*_M₁[M₂(CN)_6_]y$\Upsilon_{1}$-y·zH₂O, 0 < x < 2, 0 < y < 1), which deliver more than 150 mAh g^−1^ at 3.0 to 3.4 V versus Na^+^/Na in mildly acidic or high-concentration aqueous electrolytes and can be precipitated at room temperature, meet this requirement [5–7]. In this structure, ‘A’ represents an alkali metal, M_1_ and M_2_ are transition metals connected by cyanide ligands (C≡N), and $^{\prime}\Upsilon ^{\prime}$ signifies vacancies resulting from the absence of [M₂(CN)_6_] groups. Among the various Prussian blue analog compositions, Na_*x*_Mn[Fe(CN)_6_]·nH₂O (Mn-HCF) stands out because both Mn^2+^/Mn^3+^ and Fe^2+^/Fe^3+^ redox couples are active, giving a theoretical capacity of 170 mAh g^−1^ and an average discharge plateau above 3.2 V (vs. Na^+^/Na), making it one of the most promising cathodes for high-energy ASIBs [8, 9].

Unfortunately, structural distortions induced by sequential phase transitions and the Jahn–Teller effect jointly drive Mn dissolution, which shortens the cycling lifespan to a level that no longer satisfies practical requirements [10, 11]. Current research aimed at mitigating Mn dissolution operates along two main pathways, namely structural optimization (e.g., cation doping [12, 13], surface coating [14–16]) and electrolyte engineering [17, 18] (e.g., “water-in-salt” systems). For instance, Hu et al. [19] introduced Na₂C_4_O_4_ as a functional additive that replenishes the initial Na^+^ inventory loss and enriches a robust Na₂CO_3_-rich solid electrolyte interphase to reinforce cycling stability. Qiao et al. [20] developed a Ni/C coating that creates an H_3_O^+^-rich local environment to suppress the parasitic oxygen evolution reaction while embedding Ni atoms into the host lattice in situ, thereby maintaining structural integrity. Furthermore, Xing et al. [21] constructed a surface network on Mn-HCF using sodium alginate, where strong [Mn(SA)n] coordination bonds immobilize Mn^2+^ ions and thus retard their dissolution. While these strategies mitigate manganese dissolution to some degree, two critical limitations remain. First, most approaches focus exclusively on preventing the initial leaching of Mn or blocking its dissolution pathways yet fail to fundamentally resolve the continuous formation of fresh defects during prolonged cycling. Second, these methods widely overlook a key scientific issue: they do not examine whether dissolved Mn^2+^ introduced into the electrolyte would trigger detrimental secondary reactions, which may ultimately accelerate cell degradation and performance failure.

Herein, we unveil a previously overlooked degradation chain reaction initiated by Mn dissolution. We demonstrate that dissolved Mn^2+^ ions catalyze interfacial water oxidation, generating protons that acidify the local environment. This local pH swing subsequently promotes the decomposition of Fe(CN)_6_^4−/3−^ ligands, accelerating the structural collapse. This discovery reveals that merely suppressing the initial dissolution is insufficient; the detrimental chain reaction propagated by dissolved ions must also be severed. Based on this mechanism, we implement an in situ surface repair strategy that couples source blocking with vacancy refilling. Specifically, a concentrated 17.6 m NaClO_4_ electrolyte is used to constrain Mn loss, while trace Fe^3+^ introduced as Fe(OTf)_3_ spontaneously occupies the emerging Mn vacancies. The resulting Fe-HCF phase is thermodynamically more stable than Mn-HCF, and its closely matched lattice parameters enable coherent surface overgrowth that interrupts the dissolution chain. PTCDI is a typical n-type organic electrode material with suitable anode potential and stable structure, making it well-suited for aqueous anodes. PTCDI is chosen as the anode for its low redox potential, which pairs well with the Mn-HCF cathode to achieve a high full-cell output voltage (~ 1.4 V) and excellent electrolyte compatibility. The repaired Mn-HCF cathode exhibits a high specific capacity of 118.5 mAh g^−1^ and retains 80% of its initial capacity after 20,000 cycles at 2 A g^−1^, a stability that surpasses most previously reported manganese-based cathodes for ASIBs.

## Experimental Section

### Materials

Na_4_Fe(CN)_6_ (99%), Na_2_SO4 (99%), Mn(CH_3_COO)_2_·4H_2_O (99%), Fe(CF_3_SO_3_)_3_ (98%), and FeSO_4_·7H_2_O (99%) were purchased from Aladdin. Ascorbic acid (99%), EDTA-4Na (99%), and C_24_H_10_N_2_O_4_ (PTCDI, 98%) were purchased from Macklin. All chemicals and reagents were used as received without further purification.

### Electrode Preparation

Following a reported co-precipitation method, Mn-HCF and Fe-HCF were prepared. In a typical synthesis, 2.46 g of Na_4_Fe(CN)_6_ was dissolved in 100 mL of deionized water to form solution A. Concurrently, 1.52 g of EDTA-4Na and 1.96 g of Mn(CH_3_COO)_2_·4H_2_O were dissolved in 100 mL of deionized water to form solution B. Subsequently, solution B was slowly added dropwise into solution A at 25 °C, and the mixture was stirred for 15 min, and aged for 4 h. The resulting white precipitate was collected by centrifugation and washed several times with deionized water and alcohol. Finally, the Mn-HCF powder was obtained after drying under vacuum at 80 °C for 12 h. The synthesis process for Fe-HCF was identical to that of Mn-HCF, except that an equimolar amount of iron(II) sulfate heptahydrate (FeSO_4_·7H_2_O) was used as the starting material instead of manganese(II) acetate tetrahydrate. The cathodes and anodes were fabricated by coating a slurry onto graphite paper. The slurry was composed of the active material (Mn-HCF, Fe-HCF, or PTCDI), Super P, and polyvinylidene fluoride (PVDF) in a weight ratio of 7:2:1 or 8:1:1. The cathode films were then cut into circular electrodes with a diameter of 12 mm, while the anodes were cut into a diameter of 14 mm. Except for high-loading electrodes, which are specifically noted, the mass loading of the active material on the cathode was approximately 1–2 mg cm^−2^. For high-mass-loading electrodes, the active material, Ketjen black, and polytetrafluoroethylene (PTFE) were uniformly mixed in a mass ratio of 8:1:1. The resulting paste was then roll-pressed onto a titanium mesh. The active material loading for these electrodes was approximately 13–15 mg cm^−2^.

### Electrolytes Preparation

The WISE-17.6 m electrolyte was prepared by dissolving 17.6 mmol of NaClO_4_ in 1 mL of H_2_O. The WISE-Fe(OTf)_3_/17.6 m was prepared by adding 0.2 mmol of Fe(OTf)_3_ to the aforementioned electrolyte. Similarly, the WISE-0.05 m- Fe(OTf)_3_/17.6 m and WISE-0.1 m- Fe(OTf)_3_/17.6 m electrolytes were prepared by adding 0.05 mmol and 0.1 mmol of Fe(OTf)_3_, respectively, to the base electrolyte.

### Electrochemical Measurement

A coin-cell battery (2032) was assembled using a specified cathode, a PTCDI anode, a prepared aqueous electrolyte, and a glass fiber separator. All electrochemical measurements were carried out with a temperature of 25 °C. The electrochemical stability window of the electrolytes was evaluated using linear scan voltammetry (LSV) at a scan rate of 1 mV s^−1^, with titanium foil serving as both the cathode and anode. Cyclic voltammetry (CV) curves for Mn-HCF in both the blank and modified electrolytes were collected at a scan rate of 0.5 mV s⁻^1^. CV measurements at different scan rates were performed in a three-electrode system, with Mn-HCF as the working electrode, Pt as the counter electrode, and saturated Ag/AgCl as the reference electrode [22–24]. Electrochemical impedance spectroscopy (EIS) data were recorded over a frequency range of 0.1 Hz to 100,000 Hz. These electrochemical tests were conducted on a DH7003 electrochemical workstation. Galvanostatic charge–discharge tests and rate capability tests were performed using a Neware battery system within a voltage range of 0–2.2 V, which N/P is about 1.5. The pH of the electrolytes was measured using a Mettler Toledo PE28 pH meter. All electrochemical measurements were carried out at a temperature of 25 °C. GITT (Galvanostatic Intermittent Titration Technique) measurements were performed on a New Wei battery test system. A constant current pulse at a current density of 50 mA g^−1^ was applied for 10 min, followed by a 30 min relaxation interval to reach a quasi-equilibrium state. The pulse-relaxation procedure was repeated cyclically over the full charge–discharge voltage range. The apparent Na^+^ diffusion coefficient (D_Na_^+^) was calculated using the following equation [25–28]:

$${D}_{{Na}^{+}}=\frac{4}{\tau \pi }{(\frac{{V}_{M}{m}_{B}}{{M}_{B}S})}^{2}{(\frac{{\Delta E}_{S}}{{\Delta E}_{\tau }})}^{2}(\tau \ll {L}^{2}/{\mathrm{D}}_{{\mathrm{N}\mathrm{a}}^{+}})$$

where *V*_M_, *m*_*B*_, and *M*_*B*_ represent the molar volume, electrode mass, and molar mass, respectively, *S* represents the electrode area, ${\Delta E}_{s}$ represents the potential change in the steady state, and ${\Delta E}_{\tau }$ represents the change in potential after the pulse, respectively.

The Mn-HCF electrode was characterized using cyclic voltammetry at multiple scan rates over a potential window of 0 to 1.5 V. The peak current was found to vary linearly with the square root of the scan rates (*v*^1/2^), suggesting that the charge-storage behavior is dominated by a diffusion-controlled process.

Based on the Randles–Sevcik equation [29–31]:

$${i}_{p}=2.69\times {10}^{5}{n}^{3/2}{\mathrm{ACD}}^{1/2}{v}^{1/2}$$

the apparent diffusion coefficient D of the charge carriers was calculated from the slope of the linear *i*_*p*_-*v*^1/2^ plot. In this formula, n represents the number of electrons transferred during the redox reaction, A is the effective contact area between the electrode and the electrolyte, and *C* represents the carrier concentration within the electrode.

### Characterizations

X-ray diffraction (XRD) patterns were collected using a Smart Lab X-ray diffractometer with a Cu Kα radiation source (wavelength *λ* = 0.15405 nm). Fourier transform infrared spectroscopy (FTIR) spectra were obtained on a Vertex 70-Hyperion 3000 instrument. Raman spectra were acquired using a Renishaw in Via Raman microscope with a 758 nm laser as the excitation source. Thermogravimetric analysis (TGA) was performed on a TG209F1 analyzer (Netzsch) from 30 to 400 °C at a heating rate of 10 °C min^−1^ under a nitrogen atmosphere. The elemental composition (mass fractions of C, H, and N) of the samples was determined using a Vario EL cube elemental analyzer (Elementar). X-ray photoelectron spectroscopy (XPS) measurements were performed on a Thermo Scientific spectrometer with monochromatic Al Kα radiation. Inductively coupled plasma-atomic emission spectrometry (ICP-AES) measurements were conducted on an Optima 8300 spectrometer (PerkinElmer 8300) with the following settings: input power 1300 W, plasma flow rate 12 L min^−1^, and nebulizer flow rate 0.55 L min^−1^. The morphology of the electrodes was examined by Field Emission Scanning Electron Microscopy (SEM, Hitachi Regulus 8230). High-angle annular dark-field (HAADF) images, STEM line scan profiles, and energy-dispersive spectroscopy (EDS) data were obtained via transmission electron microscopy (TEM) using an FEI Talos F200x operating at 300 kV.

## Results and Discussion

### Mechanism of the Mn Dissolution Chain Reaction

**Fig. 1:**
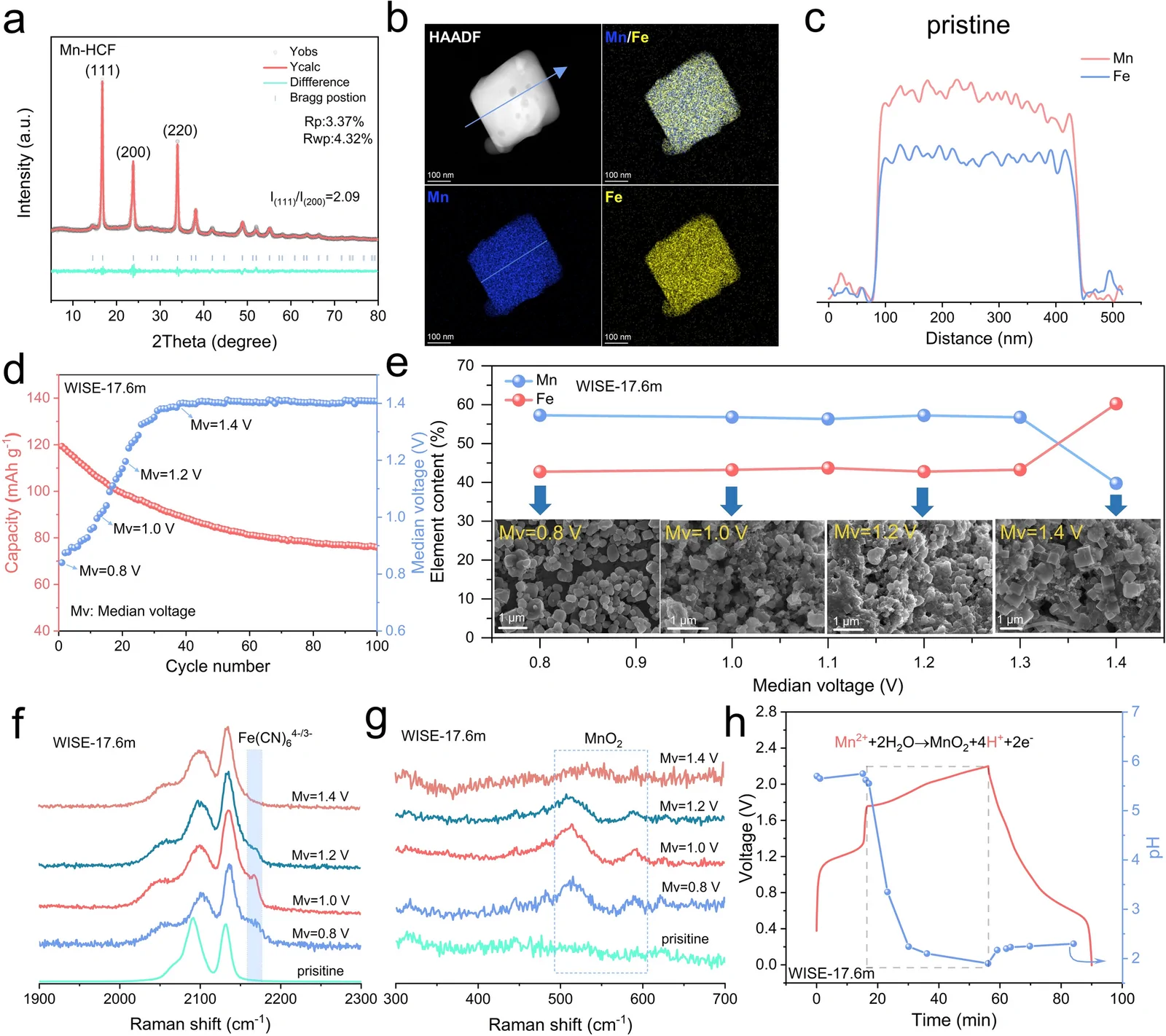
Structural characterization and electrochemical properties of Mn-HCF. **a** Rietveld refinement of the powder XRD pattern. **b, c** EDS mapping images and STEM line scan profiles. **d** Relationship between long-term cycling stability and median voltage (denoted as Mv). **e** Mn and Fe contents at different median voltages, with the inset showing SEM images at different median voltages. **f, g** Raman spectra of Fe(CN)_6_^4^⁻^/3^⁻ and MnO_2_ at different median voltages. **h** In situ pH monitoring of the WISE-17.6 m electrolyte during the initial charge–discharge cycle

The Mn-HCF and Fe-HCF samples were synthesized via a conventional co-precipitation method. As depicted in the X-ray diffraction (XRD) patterns and the corresponding Rietveld refinement plots (Fig. 1a), both samples exhibit well-defined diffraction peaks at 17.7°, 23.8°, and 33.9°, which are indexed to the (111), (200), and (220) crystallographic planes, respectively [32, 33]. The high intensity ratio of the (111) to (200) reflections (2.09:1) signifies a high degree of crystallinity. Rietveld refinement data confirm the cubic crystal structure, and the detailed crystallographic parameters are summarized in Table S1. Based on the inductively coupled plasma-atomic emission spectrometry (ICP-AES) (Tables S2 and S3), and thermogravimetric analysis (TGA) (Fig. S1) results, the molecular formulas of the as-prepared Mn-HCF and Fe-HCF were confirmed to be Na_1_0.4_7_Mn_1_0.3 [Fe(CN)_6_]·5H_2_O and Na_1.56_Fe_1_0.4_7_[Fe(CN)_6_]·4H_2_O, respectively. Transmission electron microscopy (TEM) images (Fig. 1b) show a quasi-cubic morphology with a particle size of approximately 300 nm. Furthermore, the energy-dispersive spectrometer (EDS) mapping and scanning transmission electron microscopy (STEM) line scan results (Fig. 1c) indicate the homogeneous distribution of the Mn and Fe elements. The assembled full cell using the Mn-HCF cathode and PTCDI anode exhibited a capacity retention of only 63.6% within merely 100 cycles at 1 A g^−1^ in a 17.6 m NaClO_4_ electrolyte (designated as WISE-17.6 m), with capacity decrease from 119.3 to 75.9 mAh g^−1^. As shown in Fig. 1d, along with the significant capacity decay, the discharge median voltage rapidly increases from 0.8 to 1.4 V, and the corresponding charge–discharge profiles are presented in Fig. S2. When the midpoint voltage drops to 1.4 V, the capacity decay rate slows considerably, implying that performance degradation is closely linked to structural evolution. To probe this hypothesis, the content of Mn in the cathode at different median voltage states were quantified via ICP-AES. As the median voltage increased from 0.8 to 1.2 V, the Mn content in the electrode remained almost unchanged. However, during the subsequent increase from 1.2 to 1.4 V, the Mn content decreased sharply (Fig. 1e). The ICP-AES test results (Fig. S3) indicate that Mn dissolution occurs primarily within the first 100 cycles, with a relatively fast dissolution rate. The rate of Mn dissolution was slow during the first 30 cycles (with a Mn^2+^ concentration in the electrolyte of only 0.1 mg L^−1^). After 30 cycles, the rate of Mn dissolution accelerated. By the 100th cycle, the Mn^2+^ concentration reached 0.67 mg L^−1^. After 100 cycles, the dissolution rate of Mn gradually decreased and leveled off. For iron ions, the dissolution rate remained relatively slow throughout the entire cycle. Meanwhile, the SEM images in the inset of Fig. 1e reveal that the Mn-HCF particle structure collapsed as the median voltage increase from 0.8 to 1.2 V. Paradoxically, upon reaching 1.4 V, the electrode particles recovered their nano-cubic morphology.

To disclose the relationship between the increasing median voltage and structural evolution, we performed the Raman spectra and XPS characterization of the cathodes at different voltage states. In the pristine electrode, the Raman spectra at ≈ 2090 and 2129 cm^−1^ are assigned to the stretching vibrations of the Fe^2+^–C≡N–Mn^2+^ and Fe^2+^–C≡N–Mn^3+^, respectively (Fig. 1f) [34, 35]. Meanwhile, XPS results confirm that iron exists exclusively in the divalent state in the original Mn-HCF electrode (Fig. S4a). Upon increasing the median voltage, the intensity of the Fe^2+^–C≡N–Mn^2+^ peak is significantly decreased, accompanied by the emergence of a new signal peak at 2171 cm^−1^. This phenomenon is attributed to the dissolution of Mn, which disrupts the Fe^2+^–C≡N–Mn^2+^ coordination structure and consequently exposes Fe(CN)_6_^4−/3−^ groups on the electrode surface [36]. Further XPS tests demonstrate that distinct Fe^3+^ appears when the median voltage rises to 1.4 V, with a quantified Fe^2+^/Fe^3+^ ratio of 2.3. Combined with the structural evolution described above, the generation of mixed-valence Fe species strongly confirms the formation of the Fe-HCF phase (Fig. S4b). Besides, a broadened characteristic Raman peak in the 500–650 cm^−1^ range indicates the presence of amorphous MnO_2_ in the sample (Fig. 1g) [37, 38]. EDS analysis at a median voltage of 1.2 V also shows Mn aggregation phenomena on the particle surfaces. (Fig. S5). The formation of the MnO₂ originates from the chemical reaction between dissolved Mn^2+^ and water (Mn^2+^ + 2H_2_O → MnO_2_ + 4H^+^ 2e^−^, *E*^0^ = 1.23 V vs. SHE) [39], and this dynamic dissolution–precipitation equilibrium accounts for the nearly constant Mn content in the initial stage. To further investigate the relationship between pH changes in the electrolyte and the evolution of material structure, an in situ pH study was conducted. As shown in Fig. 1h, during the charging phase, the pH initially remains stable. However, when the voltage rises to the Mn^2+^/Mn^3+^ redox plateau, Mn^2+^ begins to dissolve due to a disproportionation reaction. The dissolved Mn^2+^ catalyze interfacial water oxidation, generating hydrogen ions, which causes the pH to drop rapidly. As the extent of Mn dissolution intensifies, the acidity of the system continues to increase. Under acidic conditions, the surface Fe(CN)_6_^4−/3−^ will gradually decompose and release Fe^3+^/Fe^2+^, which explains why the Raman characteristic peak for Fe(CN)_6_^4−/3−^ completely disappears when the median voltage reaches 1.4 V. The decomposition of Fe(CN)_6_^4−/3−^ further expands the lattice defects, accelerating the dissolution of more Mn^2+^. Due to the Fe-HCF phase being thermodynamically more stable than the Mn-HCF phase, the residual Fe(CN)_6_^4−/3−^ in the system re-coordinates with the Fe^3+^/Fe^2+^ generated from decomposition to form Fe-HCF, restoring the electrode particles to their nano cubic morphology [40, 41].

**Fig. 2:**
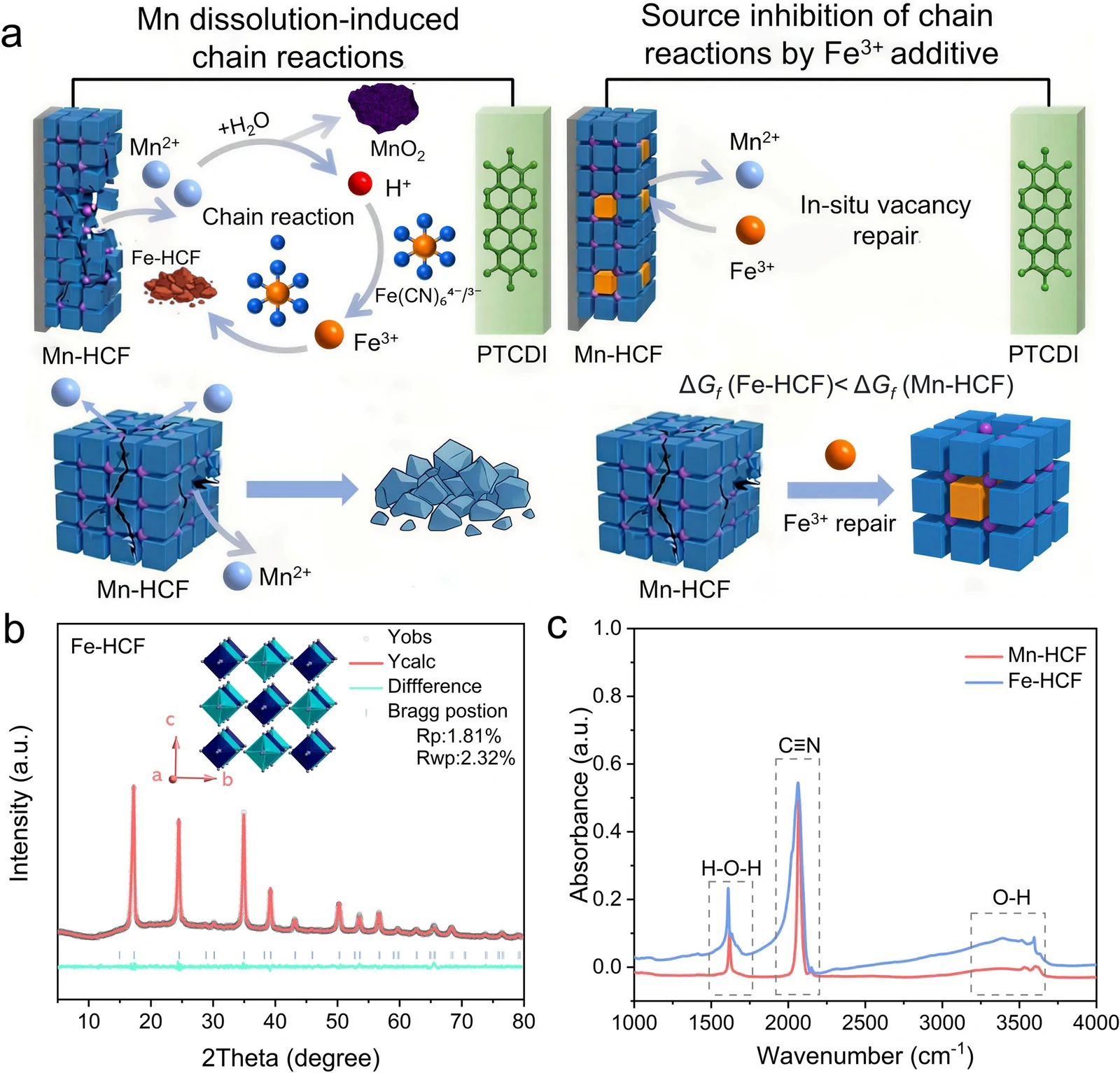
Mn dissolution-induced chain reaction, vacancy repair mechanism, and structural characterization of Mn-HCF and Fe-HCF. **a** Schematic of Mn dissolution-induced chain reaction in Mn-HCF and in situ vacancy repair mechanism by Fe^3+^ additive. **b** Powder X-ray Rietveld refinement profile for Fe-HCF, with the inset showing the crystal structure. **c** FTIR spectra of Mn-HCF and Fe-HCF

Based on the above experimental results, a chain reaction mechanism triggered by Mn dissolution is proposed (Fig. 2a). Under oxidizing conditions, dissolved Mn^2+^ can be oxidized to MnO_2_, releasing H^+^ ions. In the resulting acidic environment, Fe(CN)_6_^4−/3−^ gradually decomposes, releasing Fe^3+^/Fe^2+^. Subsequently, the remaining Fe(CN)_6_^4−/3−^ re-coordinates with the released Fe^3+^/Fe^2+^ to form Fe-HCF. As the system’s acidity further increases, MnO₂ continues to decompose back into Mn^2+^. It can be concluded that Mn dissolution triggers a series of side reactions, ultimately leading to structural collapse. Therefore, suppressing Mn dissolution is a critical strategy for improving the cycling stability of Mn-HCF cathodes. We developed a repairing agent that involves Fe^3+^ ions to rapidly occupy Mn vacancies in the Mn-HCF lattice, thereby mitigating the onset of Mn dissolution and preserving structural integrity during cycling. Fe-HCF exhibits a more negative ΔG_f_ than Mn-HCF, indicating higher thermodynamic stability and lower solubility, which drives its preferential formation at damaged Mn-HCF surfaces, thereby passivating active dissolution sites. Rietveld refinement shows that Mn-HCF and Fe-HCF are both face-centered cubic with lattice parameters of 10.50 and 10.26 Å, respectively (Fig. 2b, Table S4), corresponding to a lattice mismatch of only 2.29%. This minimal strain allows Fe-HCF to nucleate coherently on Mn-HCF surfaces, fulfilling a key geometric requirement for epitaxial repair. FTIR further reveals that the C≡N stretching modes of the two phases differ by < 1 cm^−1^ (Fig. 2c), indicating nearly identical local coordination environments and thus negligible interfacial energy penalty during the patching process.

**Fig. 3:**
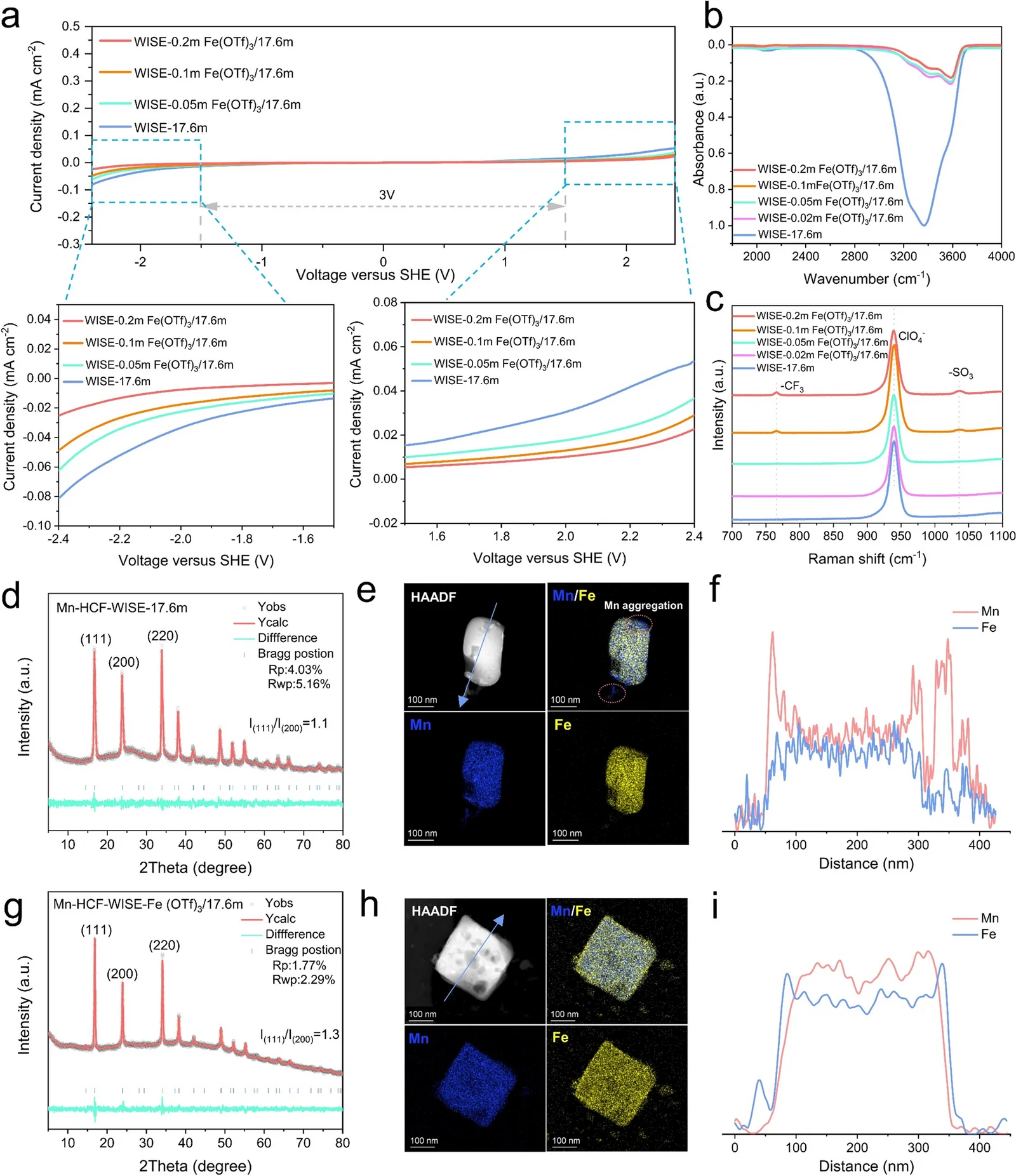
Characterizations of the Electrolyte with different Fe^3+^ concentrations and structural characterization of cycled Mn-HCF electrodes. **a** LSV curves, **b** FTIR spectra, and **c** Raman spectra. **d, g** Powder X-ray Rietveld refinement profiles of cycled Mn-HCF electrodes in WISE-17.6 m and WISE-Fe(OTf)_3_/17.6 m, respectively. EDS mapping and STEM line scan images of Mn-HCF electrode after 500 cycles in **e**, **f** WISE-17.6 m and **h**, **i** WISE-Fe(OTf)_3_/17.6 m

To investigate the effect of the Fe^3+^ additive on the electrochemical stability window (ESW) of the aqueous electrolyte, we performed the LSV measurements. “Water-in-salt” electrolytes are recognized as an effective strategy for reducing the activity of free water and broadening the ESW. As shown in Fig. 3a, the 17.6 m NaClO_4_ electrolyte enables a high ESW of up to 3 V, which is attributed to the reduced number of water molecules in the Na^+^ hydration shell, allowing ClO_4_^−^ to enter the hydration shell and interact tightly with Na^+^ [42, 43]. When the salt concentration is reduced to 8.8 M, the electrochemical stability window narrows to only 1.6 V. This not only fails to meet the voltage requirements of this full cell, but also triggers severe water splitting (Fig. S6). Meanwhile, the introduction of the Fe^3+^ additive further broadens this window, with the effect being more pronounced at higher additive concentrations. To further explore the interactions between ions and water molecules, Raman and FTIR spectra were employed (Fig. 3b, c). A 0.2 m Fe(OTf)_3_ aqueous solution shows a strong absorption peak around 3300 cm⁻^1^, indicating the presence of a large number of free or weakly hydrogen-bonded water molecules. In contrast, the 17.6 m NaClO_4_ exhibits a markedly weakened absorption peak at ~ 3300 cm⁻^1^, which is attributed to the intense competition of abundant ions for water hydrogen-bonding sites that disrupts the water network structure, thereby reducing the infrared absorption intensity of O–H vibrations. Notably, the introduction of Fe(OTf)_3_ further weakens the O–H stretching peak at ~ 3300 cm⁻^1^ with intensity decreasing distinctly upon concentration increase, indicating additive ions disrupt residual weak hydrogen bonds via competitive coordination with free water [44]. The Raman spectra show that the stretching vibration peak of ClO_4_^−^ at 938 cm^−1^ in the 17.6 m NaClO_4_ containing additives completely overlaps with that of the pure 17.6 m NaClO_4_, which demonstrates that the introduction of Fe(OTf)_3_ does not alter the primary coordination environment of ClO_4_^−^ [45, 46].

### Repair Mechanisms of Fe(OTf)_3_ Based on In Situ XRD and In Situ Raman Spectroscopy

The XRD patterns of the Mn-HCF electrodes after 500 cycles in the WISE-17.6 m and WISE-Fe(OTf)_3_/17.6 m (i.e., WISE-17.6 m with 0.2 m Fe(OTf)_3_ added) are shown in Fig. 3d, g. For WISE-17.6 m system, the I(111)/I(200) intensity ratio dropped sharply from 2.09 to 1.1, which arises from Mn^2+^ dissolution that generates abundant lattice defects, ultimately damaging the (111) crystal plane structure. In contrast, the I(111)/I(200) ratio only decreased to 1.3:1 after additive introduction, which directly confirms that Fe(OTf)_3_ can mitigate the structural degradation by repairing lattice defects, and thus enhance structural stability. Besides, EDS mapping and STEM line-scan tests were performed on both electrodes to track the elemental distribution of Mn and Fe. After long-term cycling in the WISE-17.6 m electrolyte, the reference particles exhibit severe structural damage and pronounced Mn aggregation (Fig. 3e, f). In contrast, electrodes exposed to the repairing agent display a uniform Fe-HCF coating over the Mn-HCF surface (Fig. 3h, i), corroborating that Fe^3+^-derived species epitaxially fill the vacancies created by Mn dissolution and thereby shield the underlying lattice from further decay.

To validate the efficacy of the Fe(OTf)_3_-mediated in situ repair strategy, a series of characterization techniques, including in situ pH monitoring, ex situ ICP-AES, and SEM, was employed. Figure S7 shows that the WISE Fe(OTf)_3_/17.6 m system maintains a stable pH throughout cycling which implies that Fe^3+^ suppresses Mn dissolution and thereby prevents acidification. Figure S8 tracks the Mn and Fe contents at 1 A g^−1^ where Mn decreases, while Fe increases during the first 100 cycles after which both plateau and this complementary trend indicates that Fe^3+^ continuously fills the Mn empty lattice sites during the same period. Based on the 1:1 atomic substitution relationship between Mn dissolution and Fe filling, we calculated the volume fraction of the Fe-HCF repair phase at different cycles. As shown in Fig. S9, the volume fraction of the reconstructed Fe-HCF phase reaches 15.3% after 100 cycles, and it increases most rapidly within the first 50 cycles, which is consistent with the rapid structural stabilization and suppressed polarization during the early cycling stage. The median voltage in Fig. S10 mirrors this period by dropping from 0.9 to 0.8 V within the same 100 cycles and then stabilizing which confirms that the ongoing repair process restrains further polarization. Finally, SEM images in Fig. S11 reveal an intact Mn HCF microstructure even after 1000 cycles and this corroborates that the repair strategy endows the electrode with long-term structural stability.

**Fig. 4:**
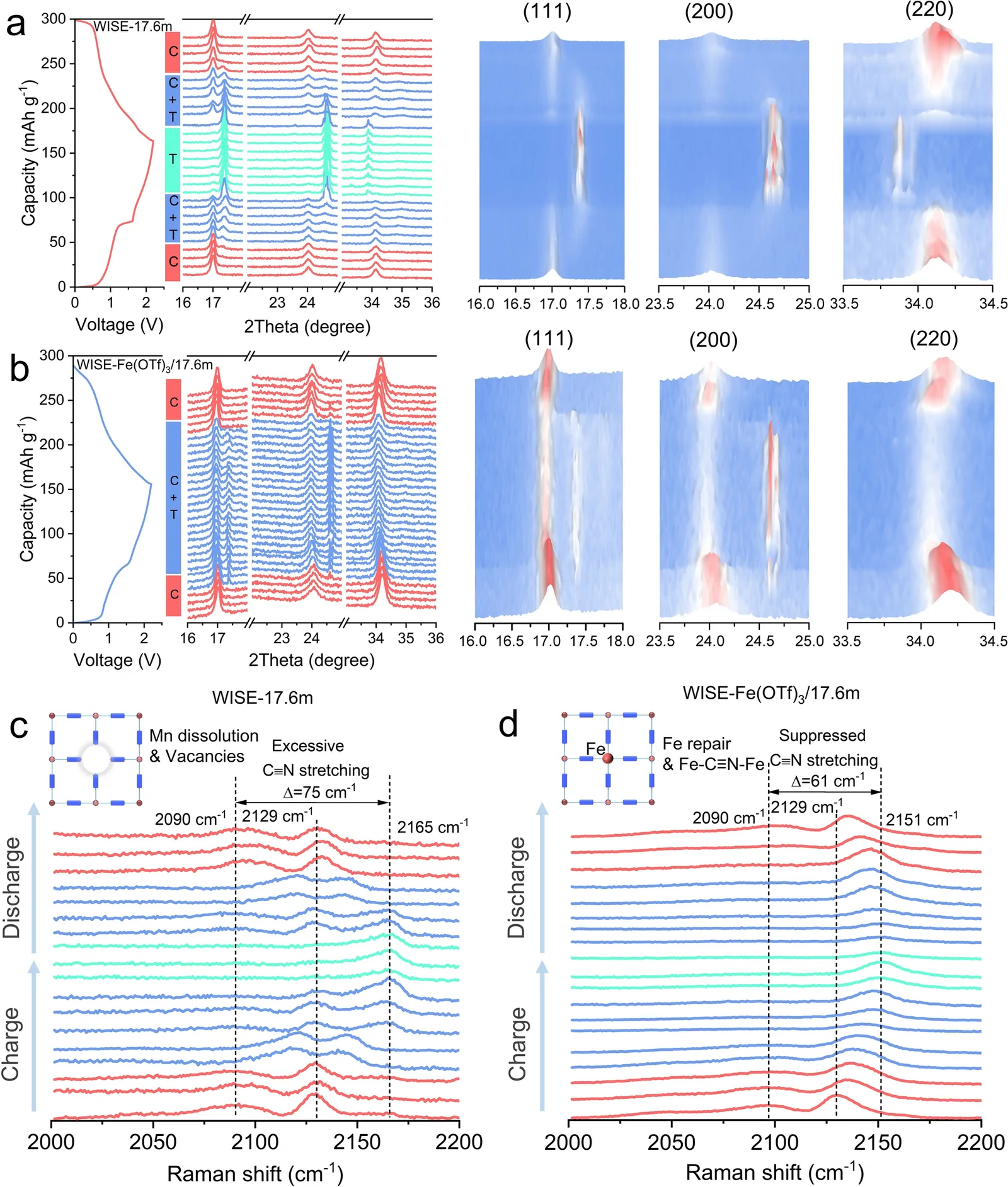
A comparison of structural evolution for Mn-HCF in WISE-17.6 m and WISE-Fe(OTf)_3_/17.6 m, respectively. **a, b** In situ XRD patterns with the corresponding charge–discharge curves and detailed in situ XRD profiles for individual crystal planes in WISE-17.6 m and WISE-Fe(OTf)_3_/17.6 m, respectively. **c** In situ Raman spectra in WISE-17.6 m, illustrating vacancy generation induced by Mn dissolution. **d** In situ Raman Spectra in WISE-Fe(OTf)_3_/17.6 m, illustrating the formation of Fe–C≡N–Fe by Fe^3+^ repair. Insets in **c, d** show the Mn dissolution and defect healing processes

To gain a deeper insight into the influence of Fe(OTf)_3_ on the structural evolution of Mn-HCF, the crystal structure during the charge–discharge process was tracked by in situ XRD. As shown in Fig. 4a, for the WISE-17.6 m system, the diffraction peaks corresponding to the (111) and (200) planes progressively shift to higher angles during charging, while the peak for the (220) plane shifts to lower angles, indicating a complete transformation of the structure from a cubic phase to a tetragonal phase. Upon discharging to 0 V, the tetragonal phase reverts to the cubic phase. This severe phase transition induces substantial volumetric strain during each cycle, which is a primary factor for particle pulverization and the degradation of long-term cycling stability. The introduction of Fe(OTf)_3_ fundamentally alters this deleterious structural evolution. As shown in Fig. 4b, during charging, the diffraction peaks at 16.8° and 24.1° split, indicating that part of the cubic Mn-HCF phase gradually transforms into the tetragonal phase. The surface layer of the particles can come into full contact with Fe^3+^ in the electrolyte. The vacancies created by the dissolution of Mn are promptly filled by Fe^3+^, leading to in situ reconstruction and the formation of a stable Fe-HCF framework. This effectively suppresses lattice distortion and maintains the cubic phase structure. However, such repair effect is limited to the particle surface. Fe^3+^ can hardly diffuse into the interior of particles, resulting in insufficient ionic repair inside the crystal. The original Mn-HCF in the interior undergoes irreversible Jahn–Teller distortion under continuous ion deintercalation and stress, gradually transforming into the tetragonal phase, ultimately leading to the coexistence of both phases [47, 48]. This restricted partial phase transformation significantly mitigates the overall volumetric deformation of the electrode and substantially enhances structural stability during long-term cycling. Meanwhile, we analyzed the in situ XRD data through Rietveld refinement (Figs. S12–S14). In the WISE-17.6 m system, the unit cell volume of the electrode expands continuously during charging, with a single-cycle volume change rate as high as 4.06%, reflecting severe structural distortion induced by Jahn–Teller effect. In contrast, the volume change rate is significantly suppressed to 2.19% in the WISE-Fe(OTf)_3_/17.6 m system. Meanwhile, the tetragonal phase ratio increases significantly with the charging voltage, reaching a maximum of 56.7% at 2.2 V, which directly quantifies the degree of cubic-to-tetragonal phase transition during the high-voltage process. During discharge, the tetragonal phase fraction decreases gradually, confirming the reversibility of this structural evolution.

In addition, we used in situ Raman spectra to follow the C≡N stretching band during charge and discharge (Fig. 4c, d). Firstly, to eliminate the influence of adsorbed water, the samples were subjected to vacuum dehydration at 120 °C. A comparison of the Raman spectra confirmed that the effect of water molecules on the C≡N peak is negligible (Fig. S15). As charging moves forward both peaks shift upward and finally overlap into one band at 2165 cm⁻^1^ at the end of charge. This merge shows that Mn^2+^ and Fe^2+^ have both turned into Mn^3+^ and Fe^3+^ giving a highly oxidized Fe^3+^–C≡N–Mn^3+^ frame. In addition, we used in situ Raman spectra to monitor the C≡N stretching band during charging and discharging (Fig. 4c, d). Compared to the initial state, the shift in the C≡N stretching vibration peak reaches 75 cm^−1^. Accompanied by the generation of Mn^3+^, severe Jahn–Teller distortion occurs throughout the lattice, inducing intrinsic internal lattice stress and framework contraction, which collectively tighten the C≡N bond and cause a distinct blue shift [49–51]. Meanwhile, lattice vacancies formed during cycling further alter the local coordination environment and bond stiffness, jointly contributing to the band shift. During the subsequent discharge, the single band at 2165 cm^−1^ slowly splits and eventually returns to two bands at 0 V, located at 2092 and 2131 cm^−1^. These two bands remain 2 cm⁻^1^ above their initial positions, suggesting irreversible structural evolution and residual vacant lattice sites after cycling. As shown in Fig. 4d, this trend differs significantly in the presence of Fe(OTf)_3_. When the battery charges to 2 V, the band only shifts to 2151 cm^−1^, with the blue shift reduced to 61 cm^−1^. This smaller shift demonstrates that the introduced Fe(OTf)_3_ effectively alleviates Jahn–Teller lattice distortion and suppresses the generation of vacancies. Meanwhile, partial Fe^3+^ occupies the Mn vacant sites and forms stable Fe–C≡N–Fe local configurations, which rigidify the cyanide framework and mitigate bond contraction. After discharge, the bands recover to 2096 and 2135 cm^−1^, 6 cm^−1^ higher than the initial positions. This difference originates from the irreversible occupation of Mn vacancies by Fe^3+^, which further enhances the structural rigidity of the surrounding coordination network.

**Fig. 5:**
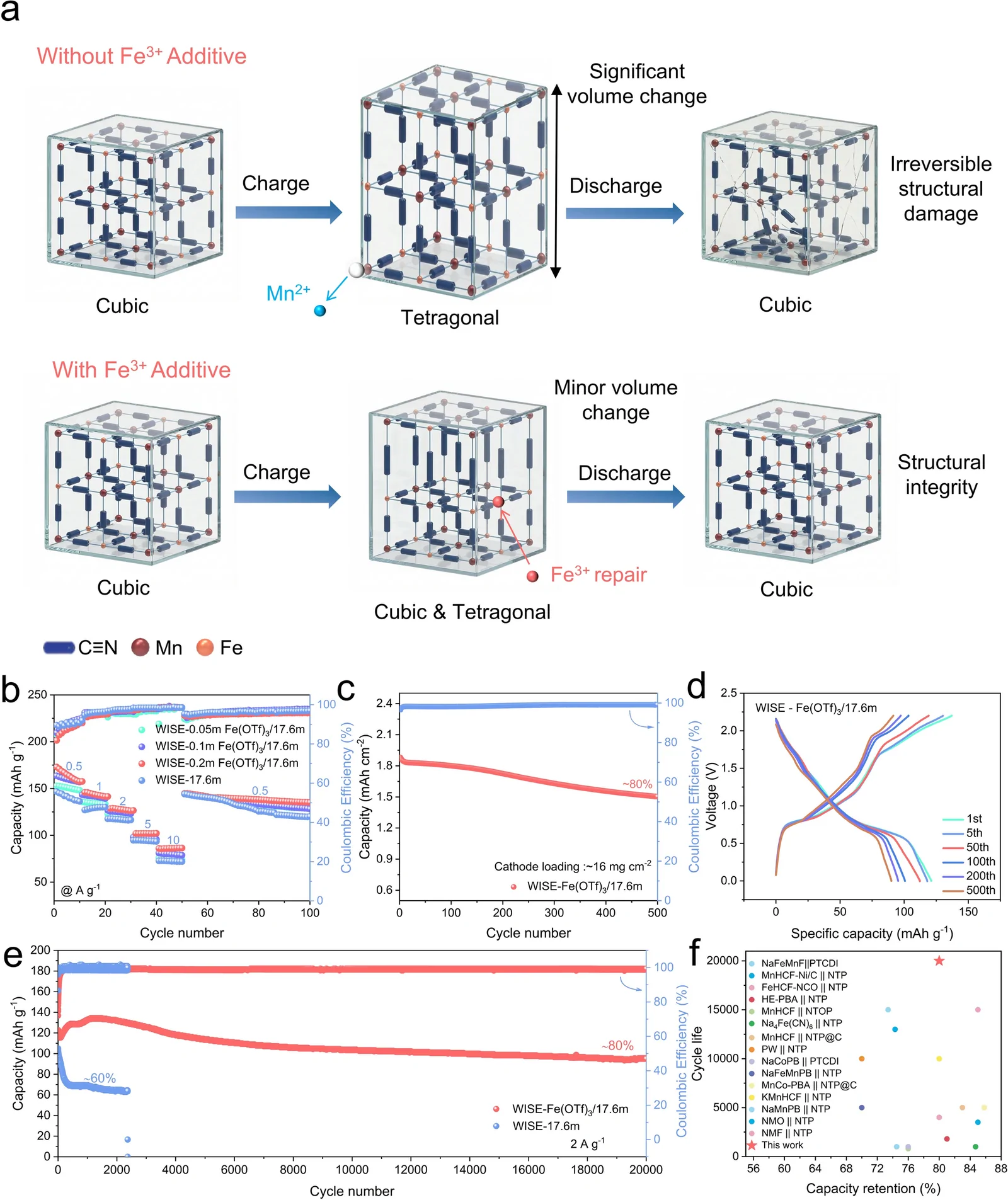
Structural changes and electrochemical performance of Mn-HCF electrodes in different electrolyte systems. **a** Comparison of the structure changes during charge/charge for Mn-HCF electrodes in different electrolytes. **b** The rate performance in blank electrolyte and modified electrolytes with different additive concentrations. **c** Cycling stability of high-loading electrode in WISE-Fe(OTf)_3_/17.6 m electrolyte. **d** Charge–discharge curves in WISE-Fe(OTf)_3_/17.6 m at different cycles at 2 A g^−1^. **e** Cycling performance of Mn-HCF electrode in WISE-17.6 m and WISE-Fe(OTf)_3_/17.6 m at 2 A g^−1^, respectively. **f** Comparison of the cycle performance between our work and those reported in the previous literature

Based on the Mn-HCF structural evolution described above we have shown that Fe(OTf)_3_ keeps the C≡N bond from stretching too far by filling Mn empty sites in situ and this helps the frame stay stable. Figure 5a illustrates this dynamic process. Mn^2+^ dissolution creates vacancies alongside the cubic-to-tetragonal transition that is already occurring. The missing ions block the return path during discharge, so the tetragonal regions cannot revert to cubic and the distortion becomes permanent. In the WISE-Fe(OTf)_3_/17.6 m system, Fe^3+^ fills the vacancies and builds an Fe–C≡N–Fe framework so the change stays local with cubic and tetragonal phases existing together and only a small volume difference. During discharge, the tetragonal regions turn back to cubic step by step, and the skeleton stays intact.

### Fe(OTf)_3_ Enhance the Electrochemical Performance of Mn-HCF Electrodes

To test the Fe^3+^ repair effect a Mn-HCF cathode and PTCDI anode full cell was assembled, and cyclic voltammetry (CV) was used to study the redox reactions. In the blank electrolyte system (Fig. S16a), the initial cycle displays two redox couples at 1.87/1.72 V and 1.21/0.61 V, corresponding to the redox reactions of Mn^2+^/Mn^3+^ and Fe^2+^/Fe^3+^. However, the Mn^2+^/Mn^3+^ redox couple weaken significantly in subsequent cycles, while the Fe^2+^/Fe^3+^ peak splits, confirming the progressive Mn dissolution and the consequent, uncontrolled formation of Fe-HCF. In the presence of Fe(OTf)_3_ (Fig. S16b), the peak shift and the decay in peak intensity are significantly reduced, demonstrating high redox reversibility. Furthermore, the Fe^2+^/Fe^3+^ redox peak also splits into two peaks, which is attributed to Fe^3+^ repairing the Mn vacancies, and the broken Fe–C≡N–Mn bonds being repaired to form Fe–C≡N–Fe bonds. Owing to its in situ repair strategy, the WISE-Fe(OTf)_3_/17.6 m system exhibits higher initial specific capacity (Fig. S17). Mn dissolution leads to capacity loss, while Fe^3+^ additives effectively repair defects, restore electrochemical activity. To investigate the effect of the Fe(OTf)_3_ additive on electrochemical kinetics, this study employs electrochemical EIS, GITT, and CV to analyze ion diffusion rates and reaction reversibility. GITT test results indicate that both sets of electrodes exhibit rapid Na^+^ diffusion capabilities, with sodium diffusion coefficients (D_Na_^+^) ranging from 10^−10^ to 10^−7^ cm^2^ s^−1^ (Fig. S18). Compared to the WISE-17.6 m system, the D_Na_^+^ values in WISE-Fe(OTf)_3_/17.6 m system were generally higher, with a particularly significant increase observed within the Mn^2+^/Mn^3+^ redox potential range. These results confirm that Fe^3+^ can optimize the structure by filling lattice vacancies, thereby accelerating sodium-ion diffusion kinetics. Furthermore, segmented GITT measurements were performed to explore the dynamic repair behavior of Fe^3+^. As displayed in Fig. S19, the D_Na_^+^ of Mn-HCF electrode gradually improved with the progression of the repair reaction. This result further confirms the in situ surface repair mechanism of Fe^3+^ from a kinetic perspective. CV measurements were conducted at different scan rates to calculate the ion diffusion coefficients, as presented in Fig. S20. The results demonstrate that the introduction of Fe(OTf)_3_ greatly increases the ion diffusion coefficients corresponding to the redox peaks of both cathode and anode. Combined with the GITT analysis, these findings further verify the positive effect of iron ion remediation on reaction kinetics. EIS reveals that the charge transfer resistance (R_ct_) in the repaired system is not only lower initially, but also continues to decrease during cycling (Fig. S21). The formation of Mn vacancies increases interfacial resistance, while Fe^3+^ additives effectively suppress this degradation pathway. Figure 5b compares the rate performance of the electrodes in the blank electrolyte and modified electrolytes. The electrode of the WISE-Fe(OTf)_3_/17.6 m system delivered discharge capacities of 168.6, 145.7, 128.8, 102.8, and 85.1 mAh g^−1^ at current densities of 0.5, 1, 2, 5, and 10 A g^−1^, respectively, significantly higher than those of the blank system (144.4, 127.0, 117.6, 94.7, and 72.7 mAh g^−1^). Meanwhile, we observed that cycling stability was enhanced with the increasing Fe(OTf)_3_ concentration. After 500 cycles, the electrolyte containing 0.2 M Fe(OTf)_3_ exhibited a higher capacity retention rate (93%) than the electrolyte containing 0.1 M Fe(OTf)_3_ (77.5%) and the blank electrolyte (49.9%). (Fig. S22).

A long-cycle performance test was further conducted at a current density of 2 A g^−1^ (Figs. 5d, e and S23). Mn-HCF electrode delivers an initial discharge capacity of 118.5 mAh g^−1^ with a capacity retention rate of 80.0% after 20,000 cycles, which is considerably higher than the 60.9% retention rate of the blank system after 2,000 cycles. Notably, the continuous capacity increase in the early cycling stage can be attributed to the vacancy-repair effect of Fe^3+^, which stabilizes the framework while in situ forming highly conductive Fe-HCF, thereby improving the electronic transport performance of the electrode [52]. The corresponding charge–discharge curves reveal that the Mn-HCF electrode with WISE-17.6 m exhibits rapid capacity decay accompanied by an increase in the median voltage. Meanwhile, the electrode with WISE-Fe(OTf)_3_/17.6 m demonstrates excellent reversibility, attributed to Fe^3+^ repairing vacancies. Even when the electrode mass loading increased to 16 mg cm^−2^, the repair strategy remained effective (Fig. 5c). At 1 A g^−1^, the electrode discharged a capacity of 1.88 mAh cm^−2^, retaining 80% (1.51 mAh cm^−2^) after 500 cycles. The Mn-HCF electrode with WISE-Fe(OTf)_3_/17.6 m also demonstrates an excellent rate performance. Specifically, the specific capacity reached 139, 120, and 86 mAh g^−1^ at 0.3, 0.5, and 2 A g^−1^, respectively (Fig. S24). The data above have confirmed that Fe(OTf)_3_ effectively alleviates the capacity decay of Mn-HCF caused by Mn dissolution and suppress the progress of the chain reaction. Relative to previously reported Prussian-blue cathode full cells (Fig. 5f, Table S5), the battery developed in this study shows higher discharge capacity and longer cycle life.

To test if the benefit of Fe(OTf)_3_ is not limited to Mn-HCF, we also built a full cell with Fe-HCF cathode and PTCDI anode and evaluated its performance. As shown in Fig. S25, the discharge capacity of the Fe-HCF electrode with WISE-17.6 m gradually decays from an initial 99.5 to 66.8 mAh g^−1^ after 500 cycles at 1 A g^−1^, with a capacity retention of 67.2%. In contrast, in the WISE-Fe(OTf)_3_/17.6 m system, the discharge capacity electrode first increases to 103.9 mAh g^−1^ and then enters a stable cycling phase, maintaining a capacity retention of 75.2% even after 500 cycles. The molar ratio of Fe to Na in the electrodes revealed that the loss rate of iron ions was significantly slowed after the addition of Fe(OTf)_3_ (Fig. S26). By comparing the SEM images after 0 (initial state), 200, and 500 cycles, the Fe-HCF particles continuously fracture and aggregate during cycling due to the influence of iron ion dissolution, leading to the loss of active material and the deterioration of electrochemical performance (Fig. S27). Under the influence of Fe(OTf)_3_, the morphology and structure of the Fe-HCF particles remain relatively complete, effectively suppressing the dissolution of iron ions. Fe(OTf)_3_ was added at a low concentration (≤ 0.2 M), costing less than 5% of the NaClO_4_-based electrolyte. It effectively inhibited Mn dissolution and structural degradation, greatly extending the cycle life with excellent cost effectiveness. As an electrolyte additive, Fe(OTf)_3_ hardly affects the cell energy density. It stabilized long-term capacity by suppressing low-activity tetragonal phase formation. In addition, the low additive dosage only slightly influences electrolyte viscosity and low-temperature performance. The above results convincingly demonstrate that the Fe^3+^-mediated in situ repair is a broadly applicable and highly effective strategy for mitigating transition metal dissolution and enhancing the stability of the wider family of PBA cathode materials.

## Conclusion

In summary, this work demonstrates that Mn dissolution is not merely a loss of active material but the trigger of a chemical chain reaction that actively destroys the cathode structure. This understanding calls for a stabilization strategy that moves from passive reinforcement to active and targeted correction at the atomic scale. Our in situ repair approach which uses trace Fe^3+^ to occupy vacancy sites suppresses this degradation pathway at its origin and delivers an order of magnitude improvement in the cycle life of both Mn-HCF and Fe-HCF full cells. The study thus provides a critical insight into a common failure mode in Prussian blue analogs and offers a generalizable design principle for achieving ultra-stable electrodes in aqueous batteries.

## Supplementary Information

Below is the link to the electronic supplementary material.

Supplementary file1 (DOCX 27622 KB)
