# Supplementary material for: In Situ Defect Healing Suppresses Mn Dissolution Chain Reactions in Aqueous Sodium-Ion Cathodes
Source: Nanomicro Lett. 2026 Jun 30;18:420. doi: 10.1007/s40820-026-02271-z (PMC13319298; doi:10.1007/s40820-026-02271-z)
Supplement: Supplementary file 1 — Supplementary file1 (DOCX 27622 KB) [file 40820_2026_2271_MOESM1_ESM.docx]

Supporting Information for

**In-Situ Defect Healing Suppresses Mn Dissolution Chain Reactions in Aqueous Sodium-Ion Cathodes**

Wenqing Du^1^, Lin Xu^1^, Yi Yang^1^, Jingying Sun^2^, Gongzheng Yang^1^*, Chengxin Wang^1^*

^1^ School of Materials Science and Engineering, State Key Laboratory of Optoelectronic Materials and Technologies, Sun Yat-sen (Zhongshan) University, Guangzhou 510275, People’s Republic of China

^2^ Instrumental Analysis and Research Center, Sun Yat-sen (Zhongshan) University, Guangzhou 510275, People’s Republic of China

*Corresponding authors. E-mail: [wchengx@mail.sysu.edu.cn](mailto:wchengx@mail.sysu.edu.cn) (Chengxin Wang); [yanggzh5@mail.sysu.edu.cn](mailto:yanggzh5@mail.sysu.edu.cn) (Gongzheng Yang)

**Supplementary Figures and Tables**


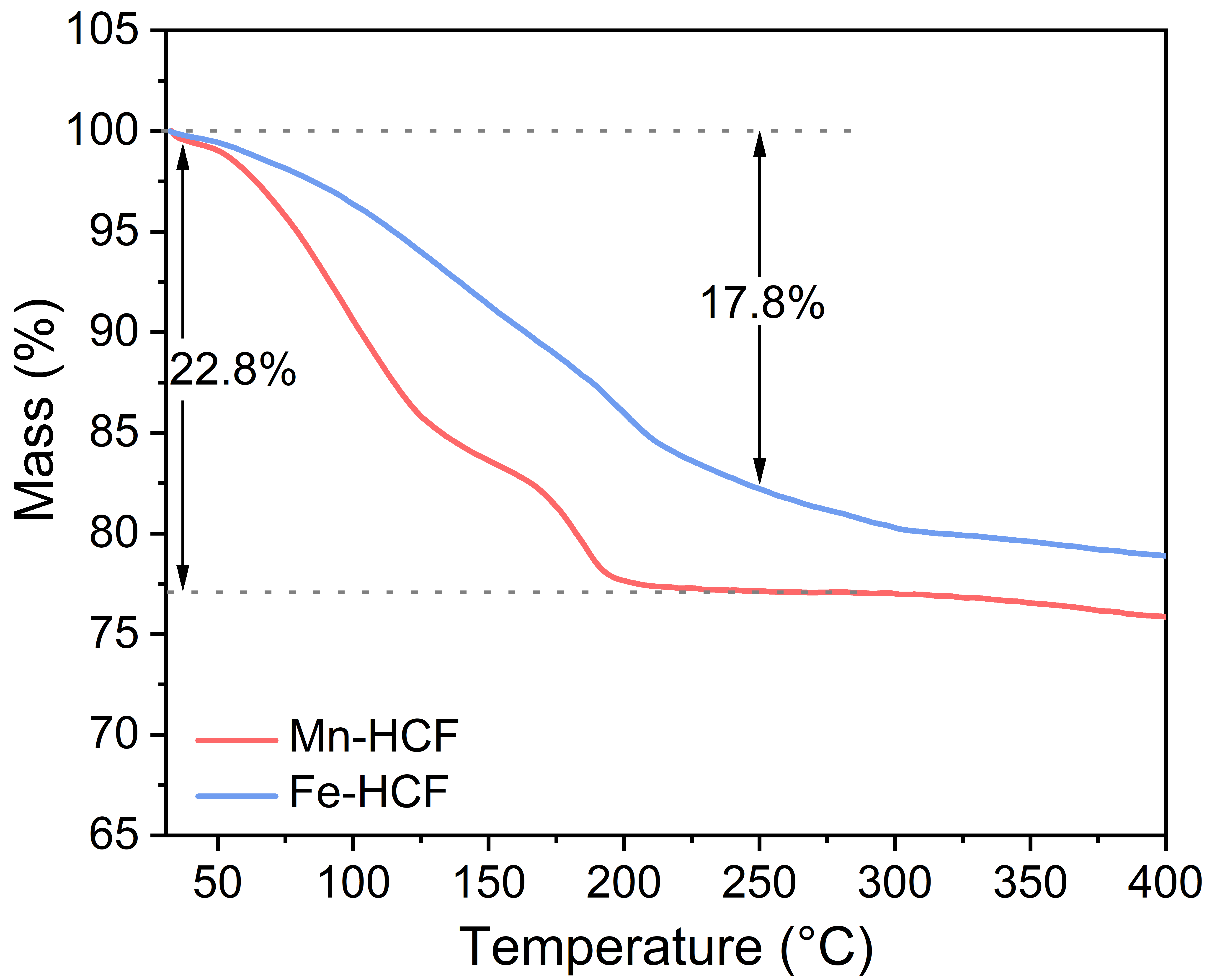


**Fig. S1** TGA results of Mn-HCF and Fe-HCF samples.


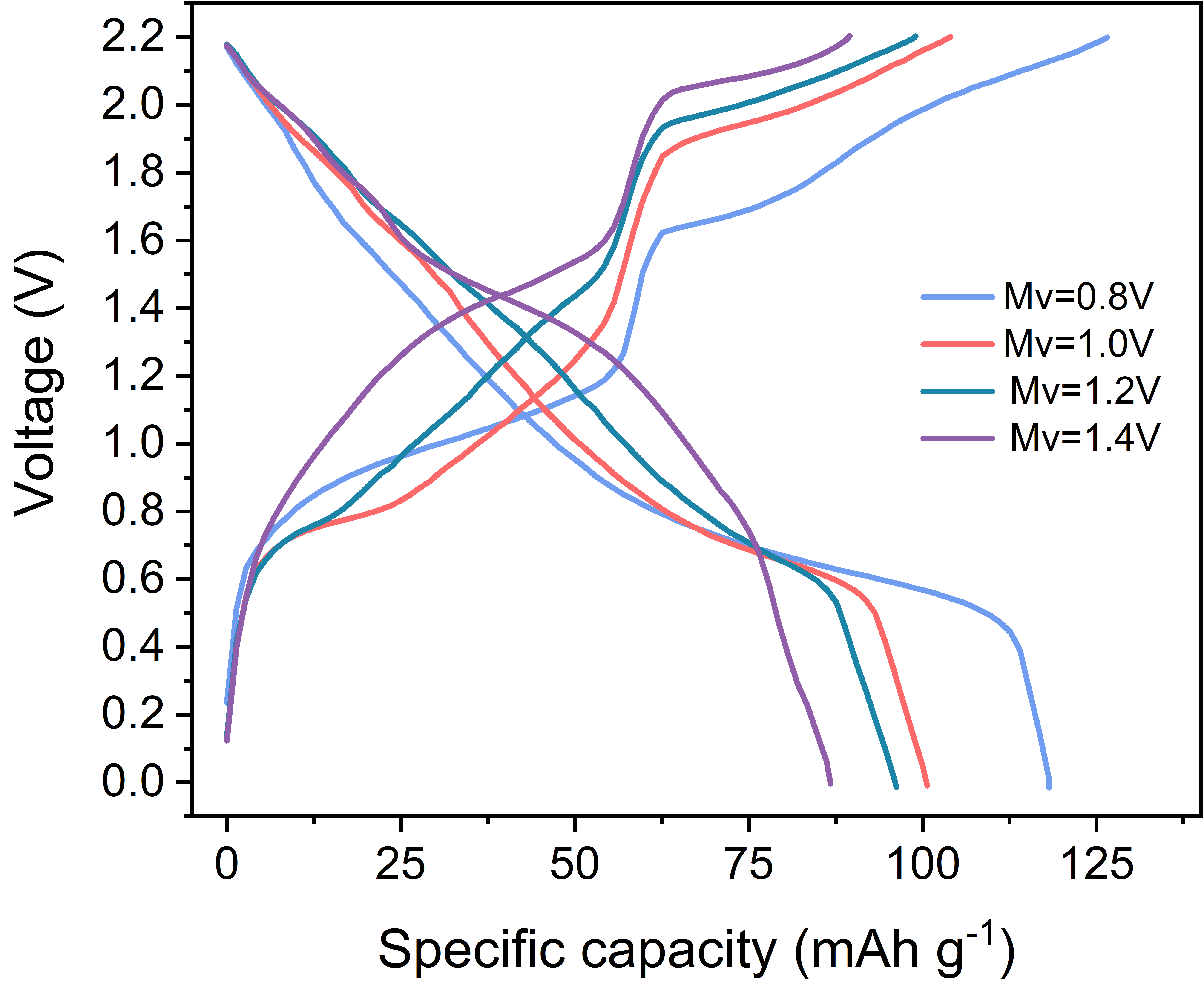


**Fig. S2** Charge-discharge curves of Mn-HCF electrodes in the voltage range from 0.8 to 1.4 V.


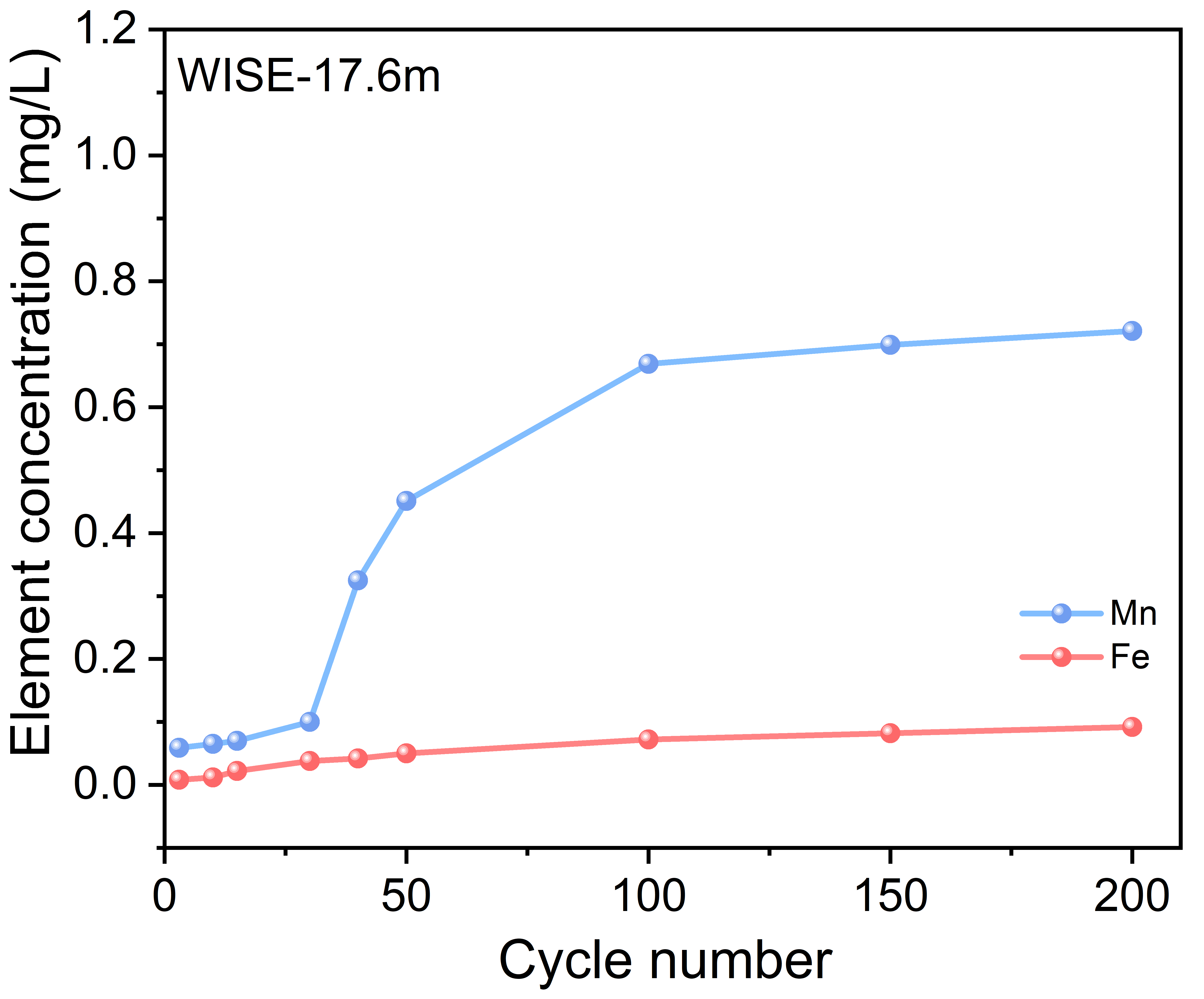


**Fig. S3** The concentration changes of Mn and Fe in the WISE-17.6m electrolytes obtained at different cycle numbers.


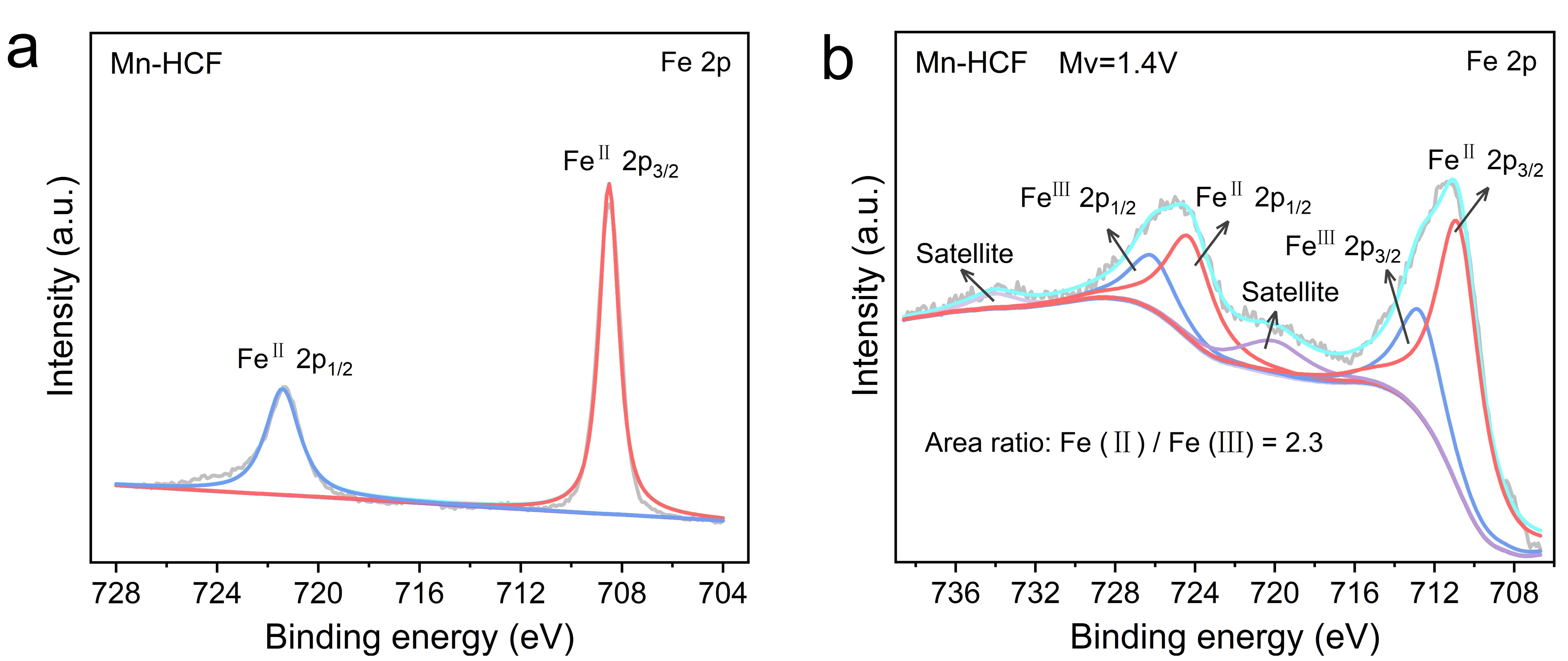


**Fig. S4** Fe 2p spectra of the (**a**) pristine Mn-HCF electrode and (**b**) the Mn-HCF electrode with median voltage at 1.2 V.


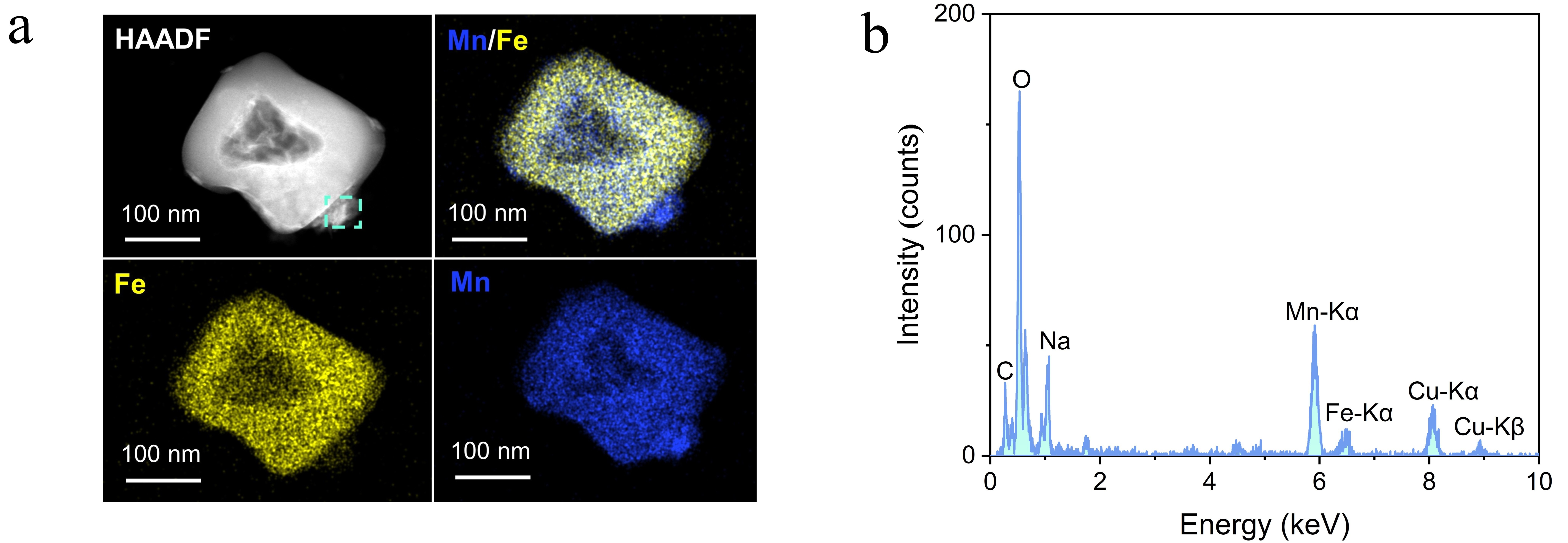


**Fig. S5** (**a**) EDS mapping and (**b**) local energy spectra of electrode particles with median voltage at 1.2 V.


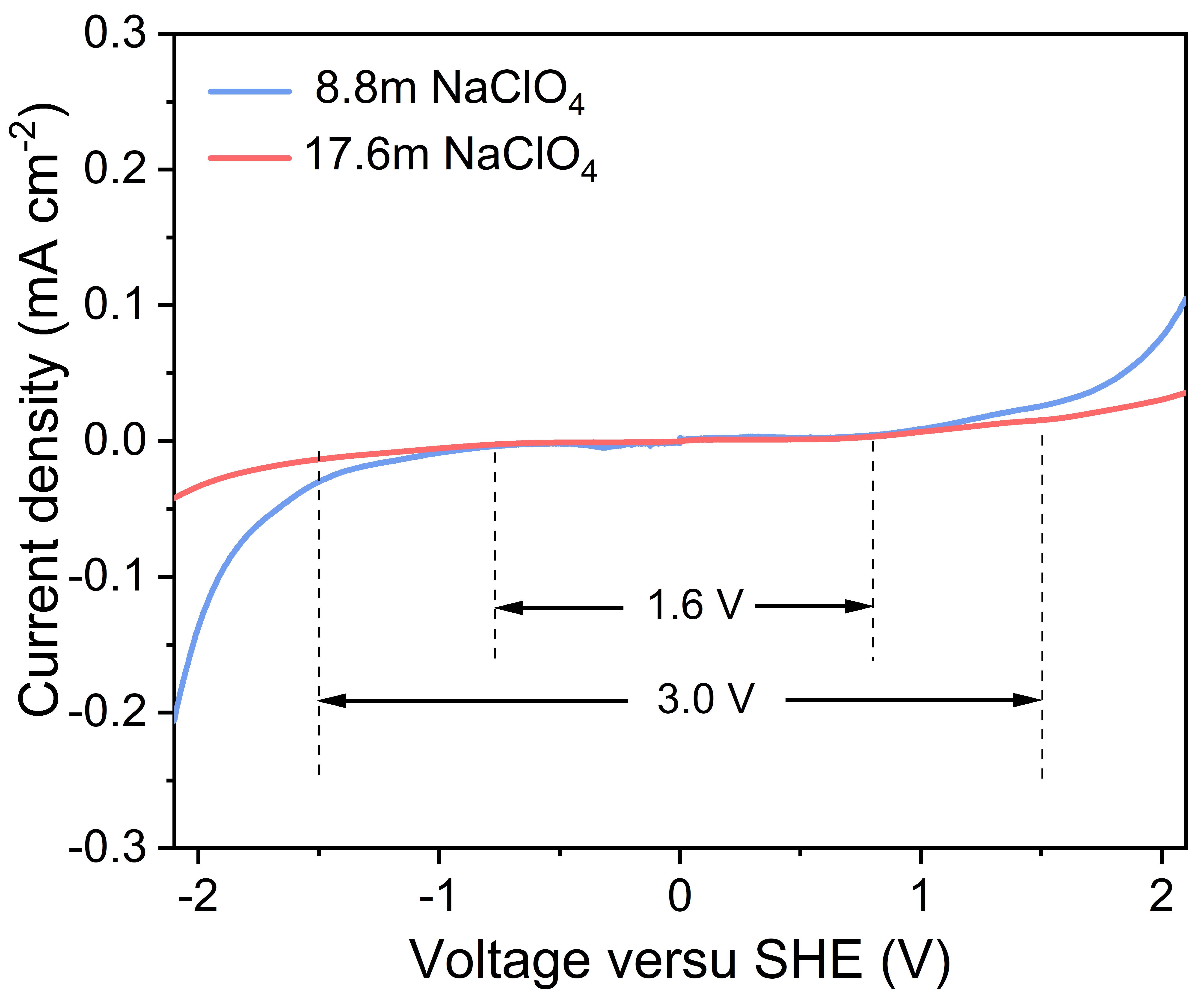


**Fig. S6** LSV curves of 8.8 M and 17.6 M NaClO_4_.


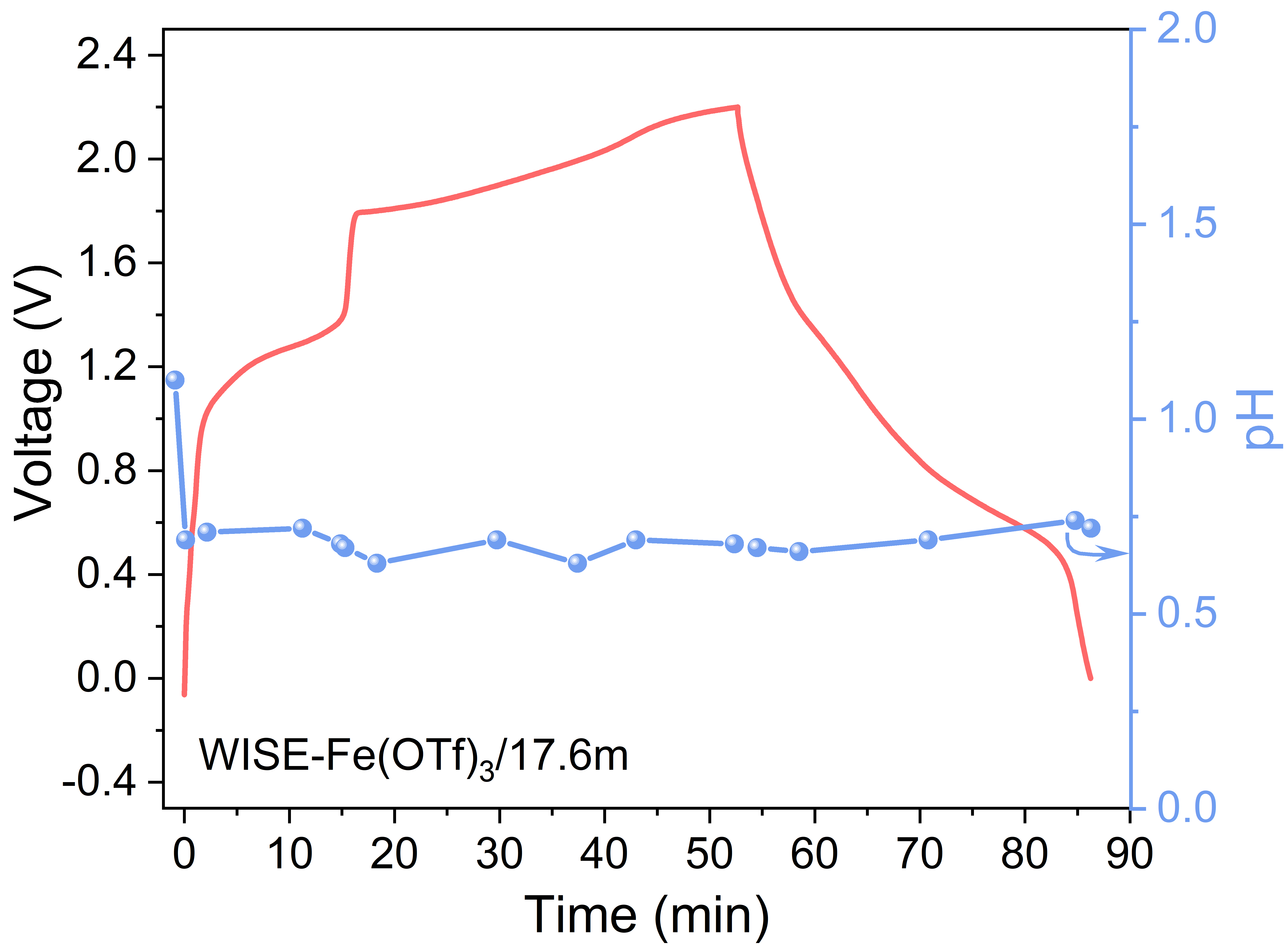


**Fig. S7** In-situ pH monitoring of Mn-HCF electrodes during the initial charge-discharge cycle in the WISE-Fe(OTf)₃/17.6m.


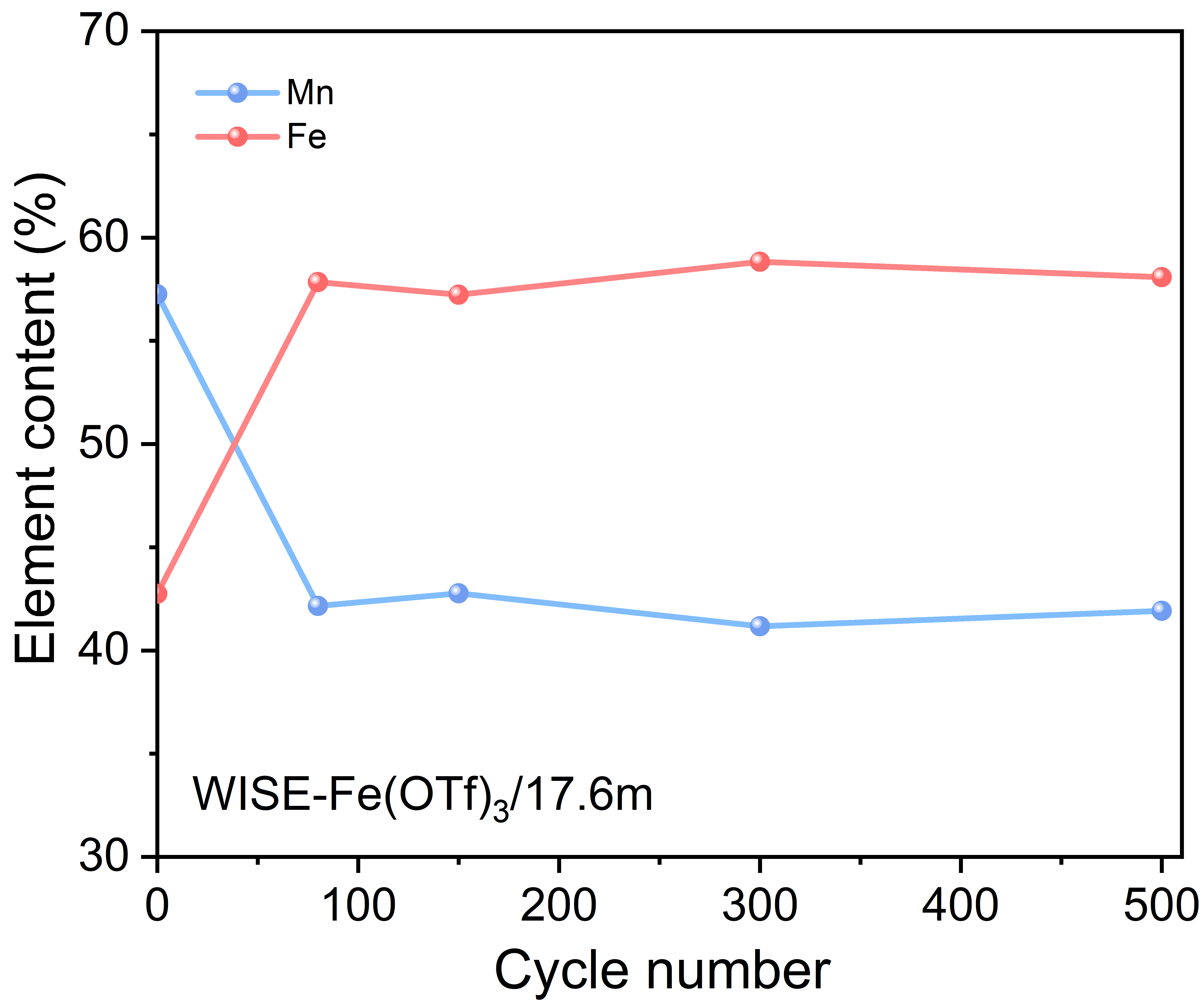


**Fig. S8** Variation in Mn and Fe content in Mn-HCF electrodes at different cycle numbers within the WISE-Fe(OTf)₃/17.6m.


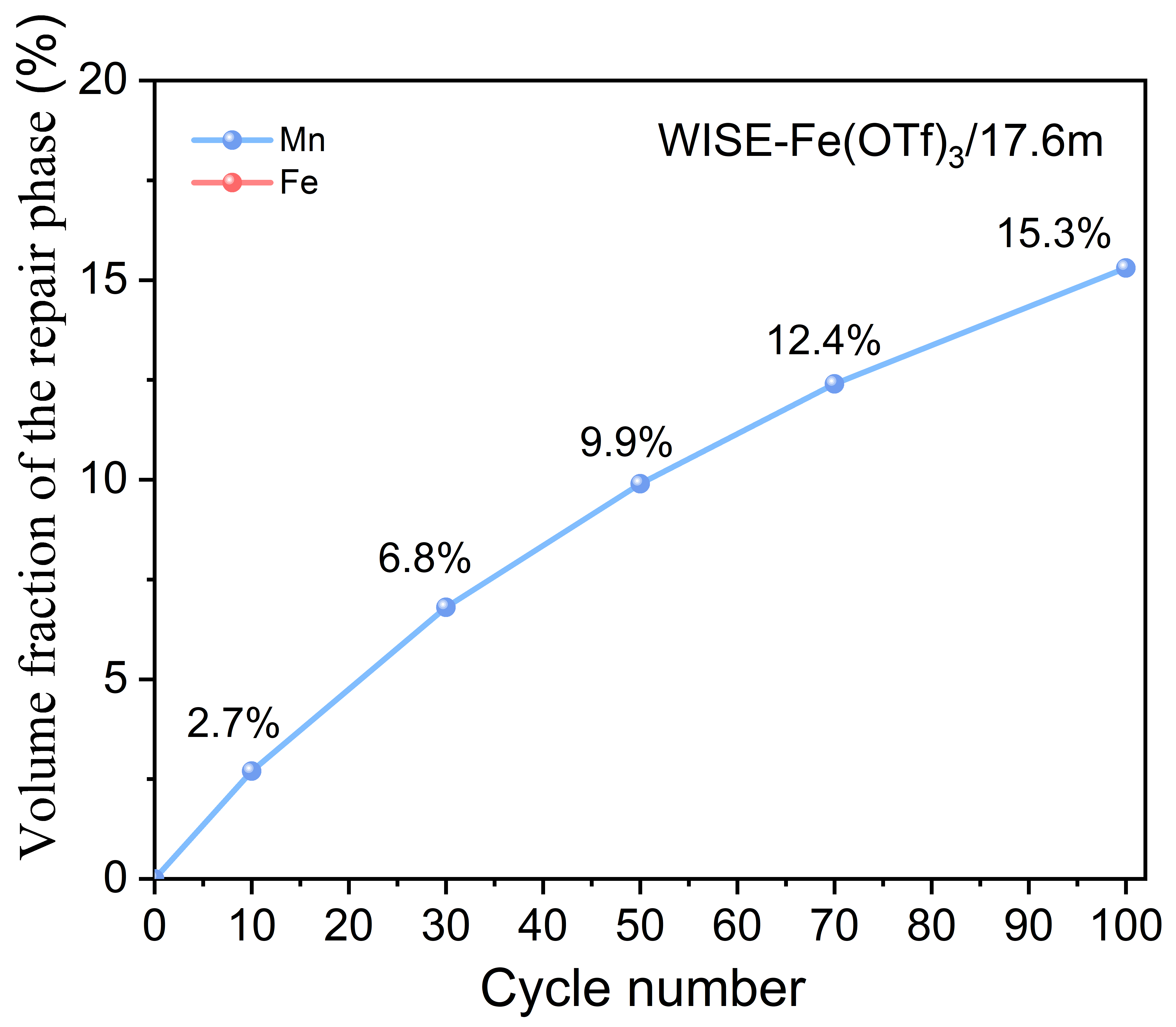


**Fig. S9** Volume fraction of the repaired phase at different cycle numbers.


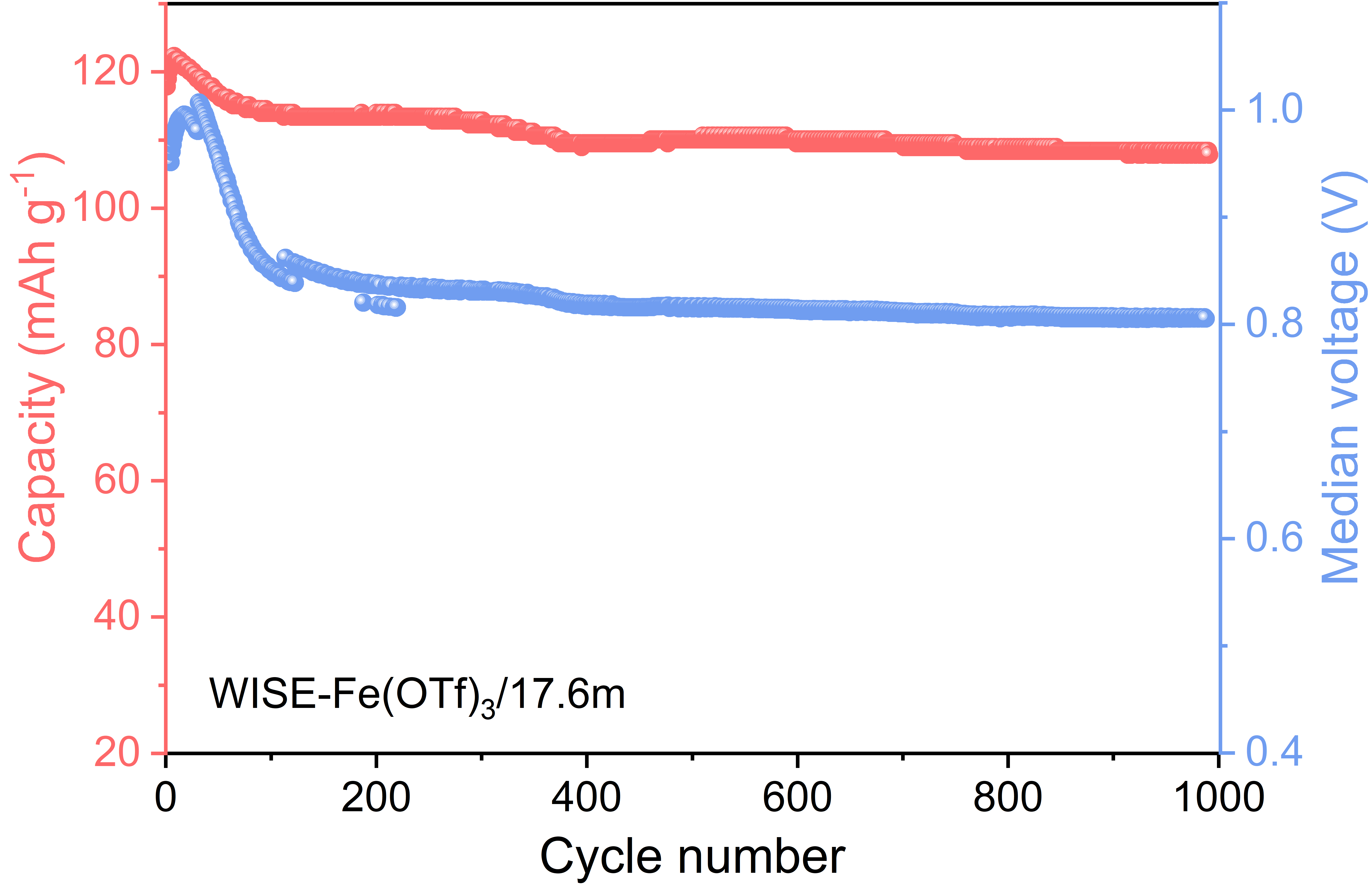


**Fig. S10** Long-term cycling stability of Mn-HCF electrodes versus median voltage in WISE-Fe(OTf)₃/17.6m.


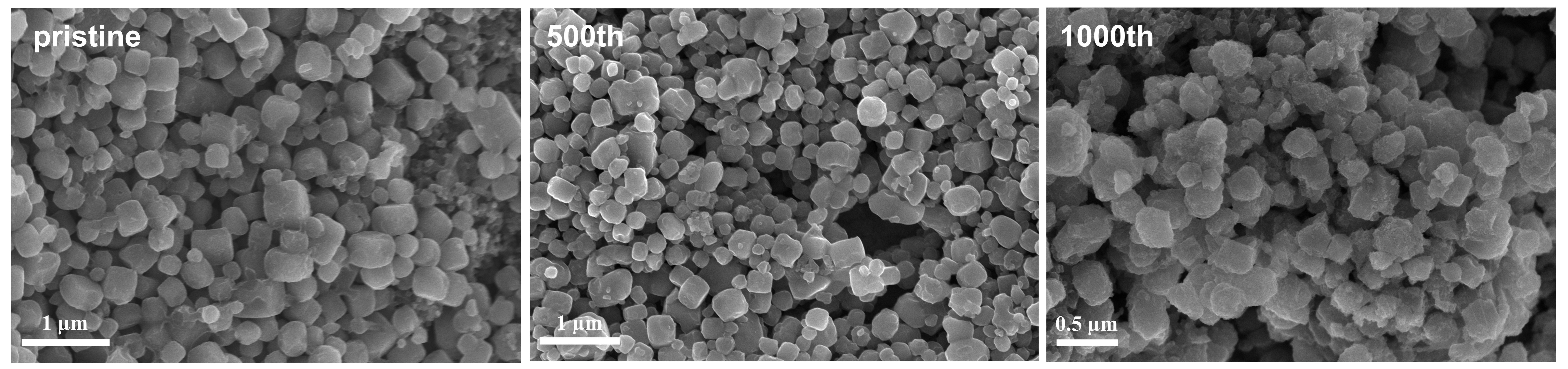


**Fig. S11** SEM images of Mn-HCF electrodes at different cycle numbers in WISE-Fe(OTf)₃/17.6m.


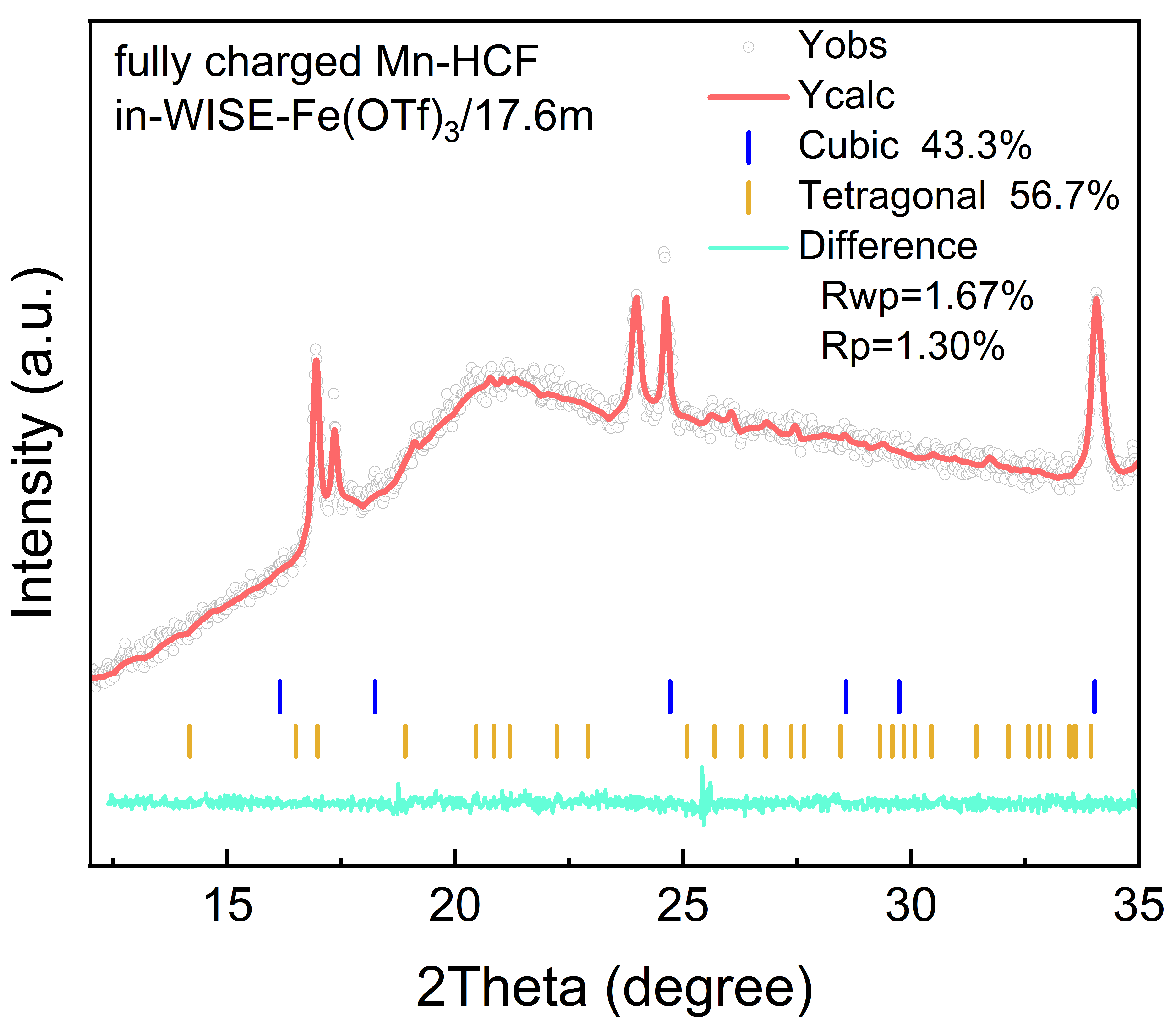


**Fig. S12** Powder X-ray Rietveld refinement profile of Mn-HCF electrodes in the fully charged state in WISE-Fe(OTf)_3_/17.6m.


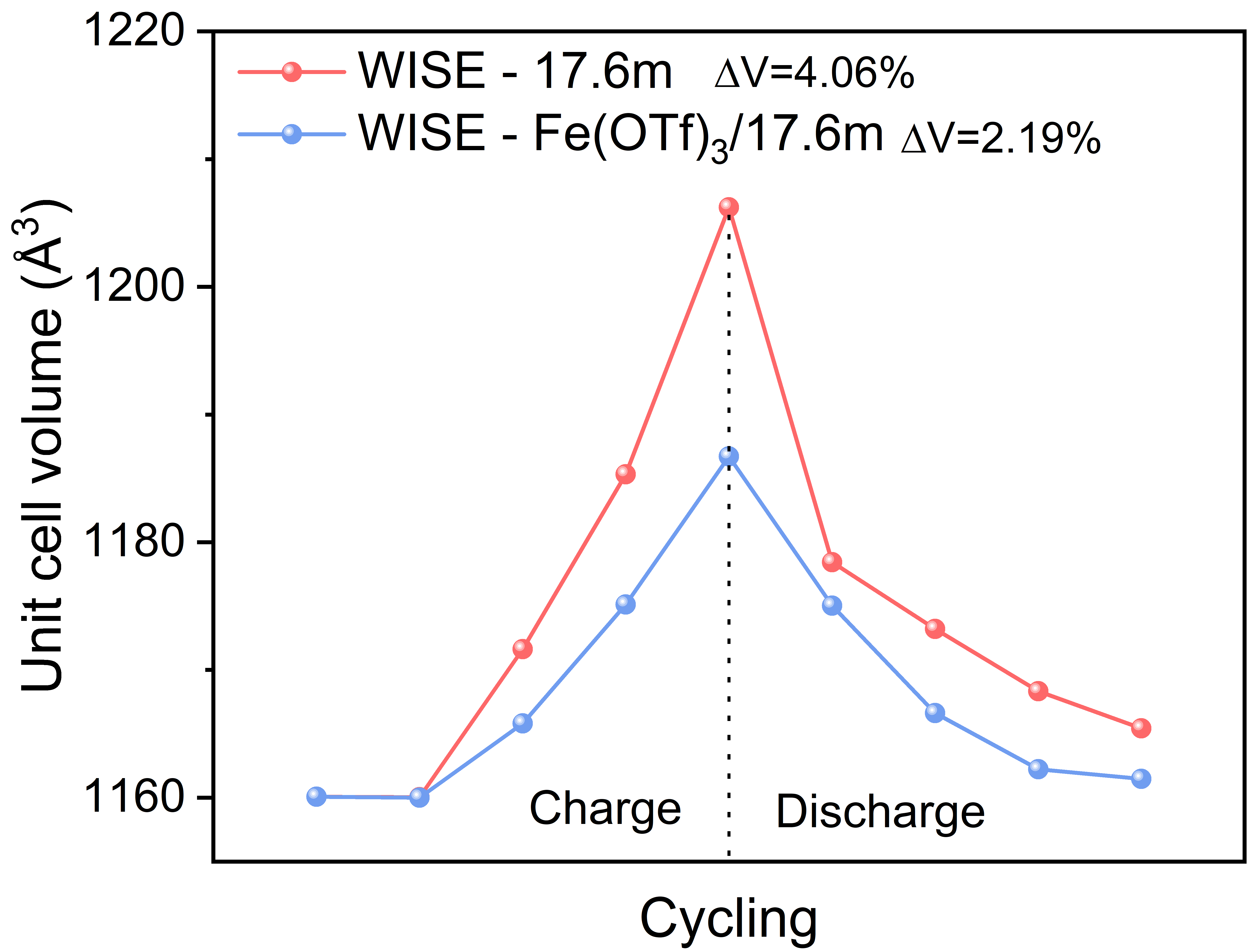


**Fig. S13** Volume changes of Mn-HCF electrode during charging and discharging in WISE-17.6m and WISE-Fe(OTf)_3_/17.6m, respectively.


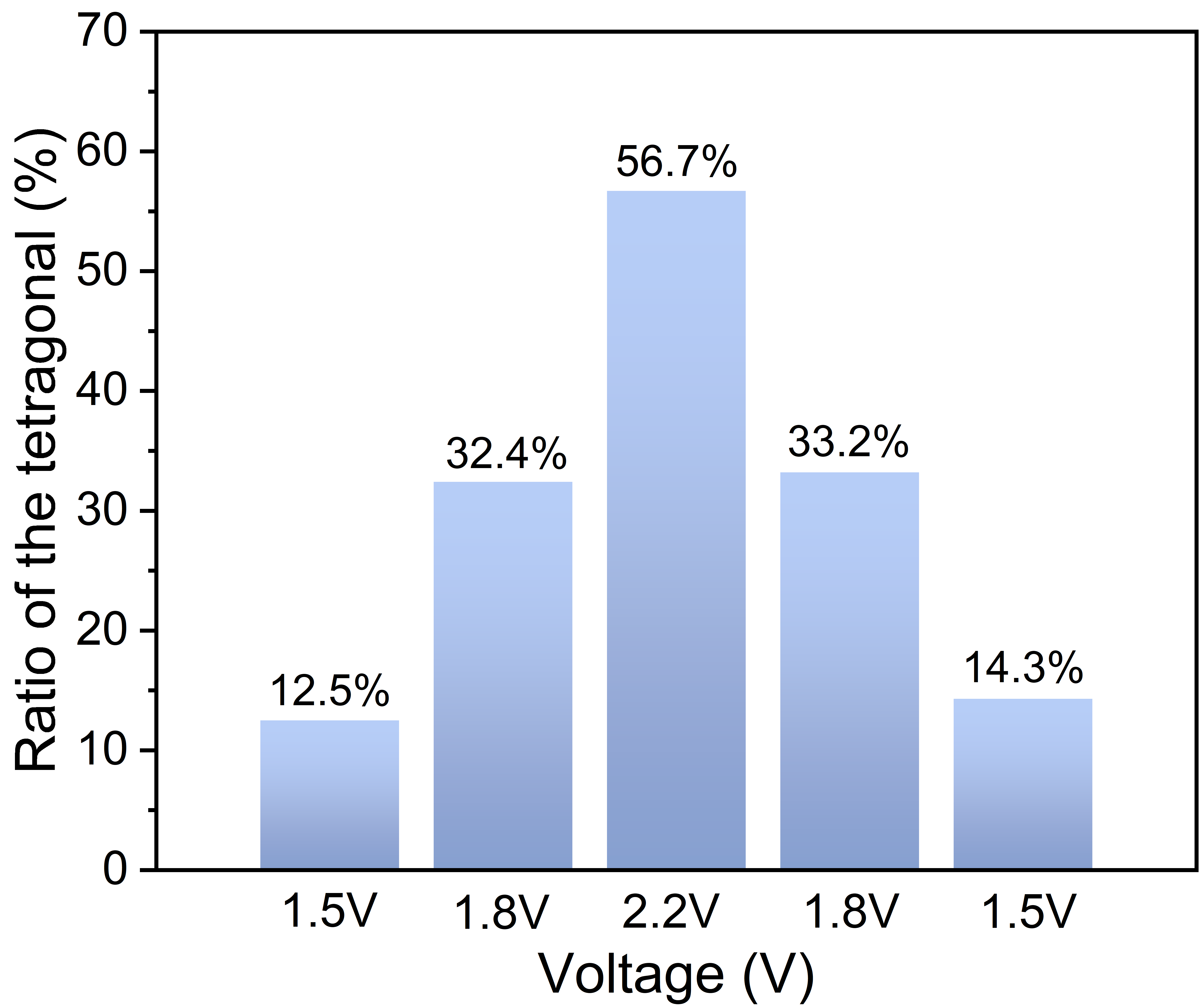


**Fig. S14** The proportion of the tetragonal phase of Mn-HCF electrode during charging and discharging in WISE-Fe(OTf)_3_/17.6m.


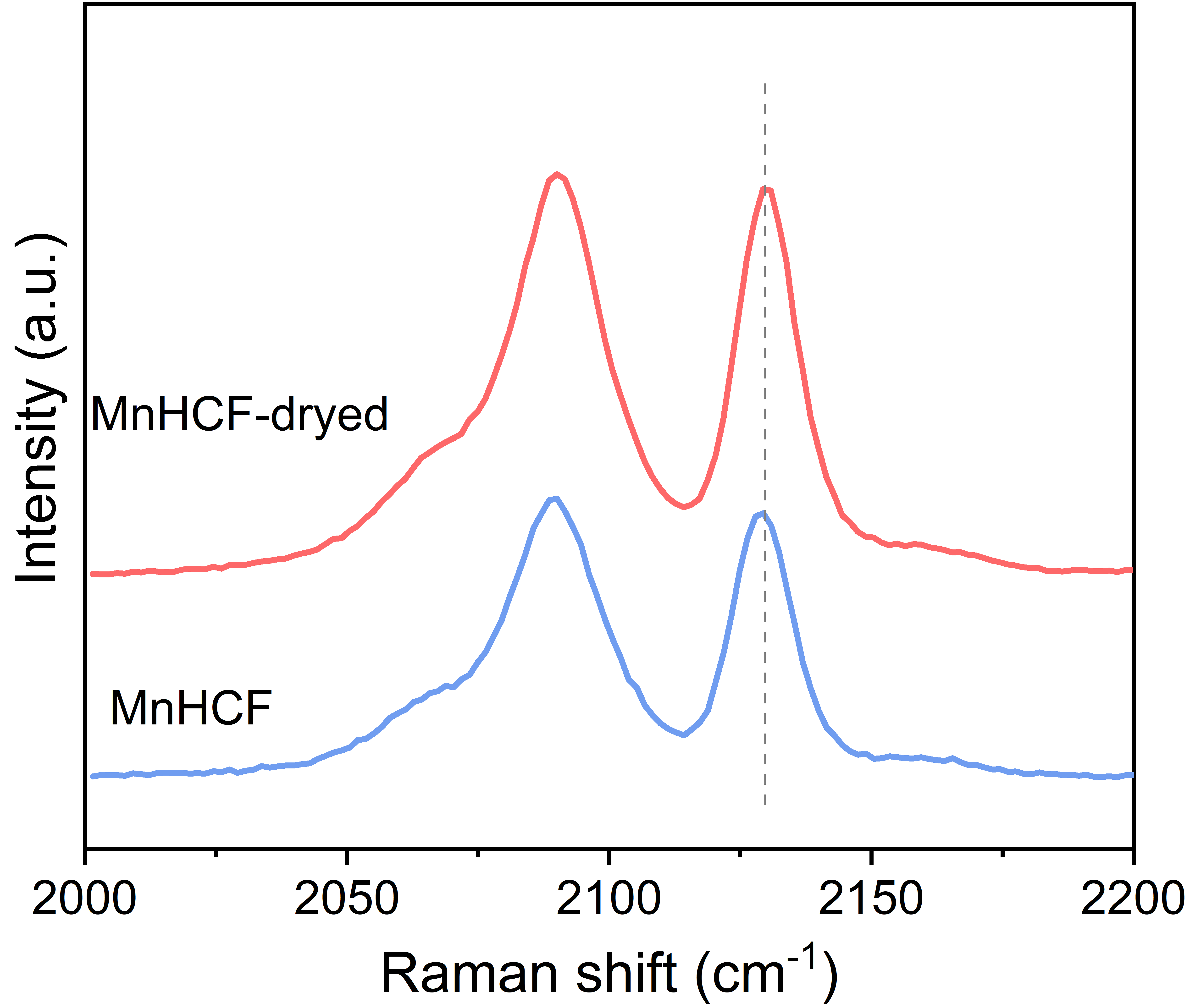


**Fig. S15** Raman spectra of the Mn-HCF electrode before and after vacuum drying at 120°C.


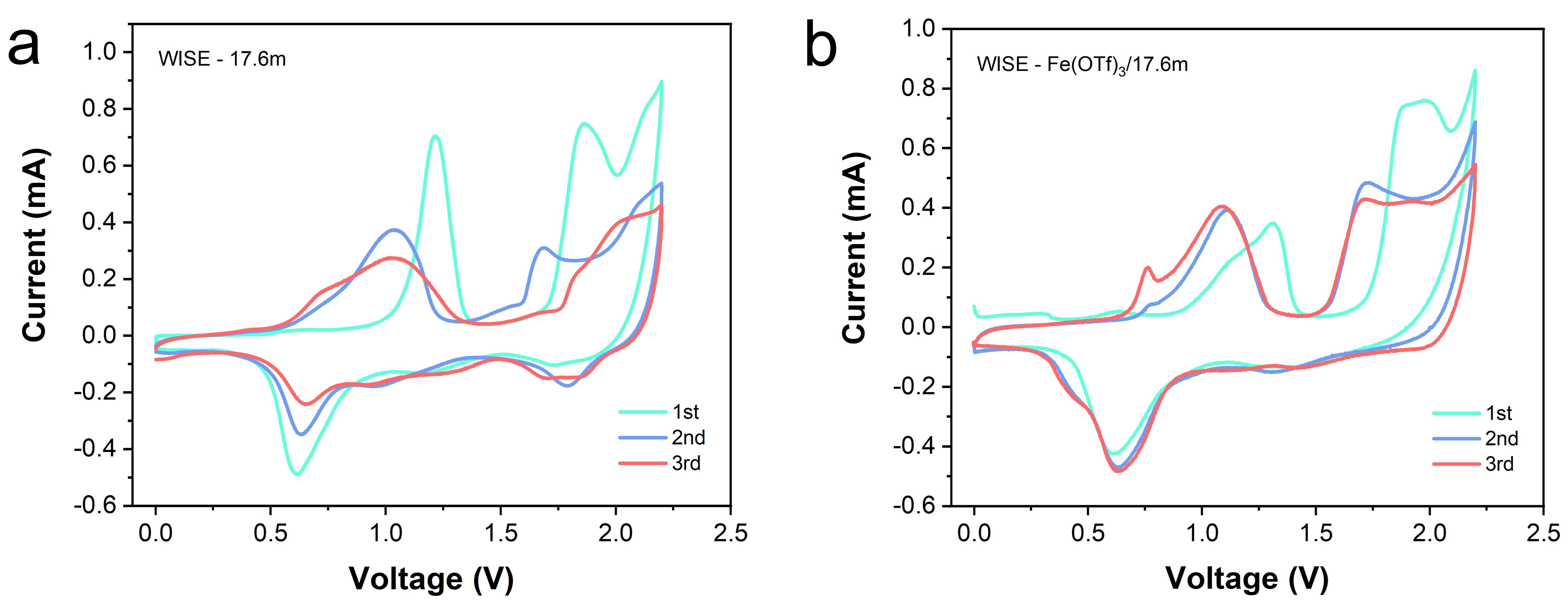


**Fig. S16** Cyclic voltammetry curves of Mn-HCF electrodes in (**a**) WISE-17.6m and (**b**) WISE-Fe(OTf)_3_/17.6m at 0.5 mV s^-1^, respectively.


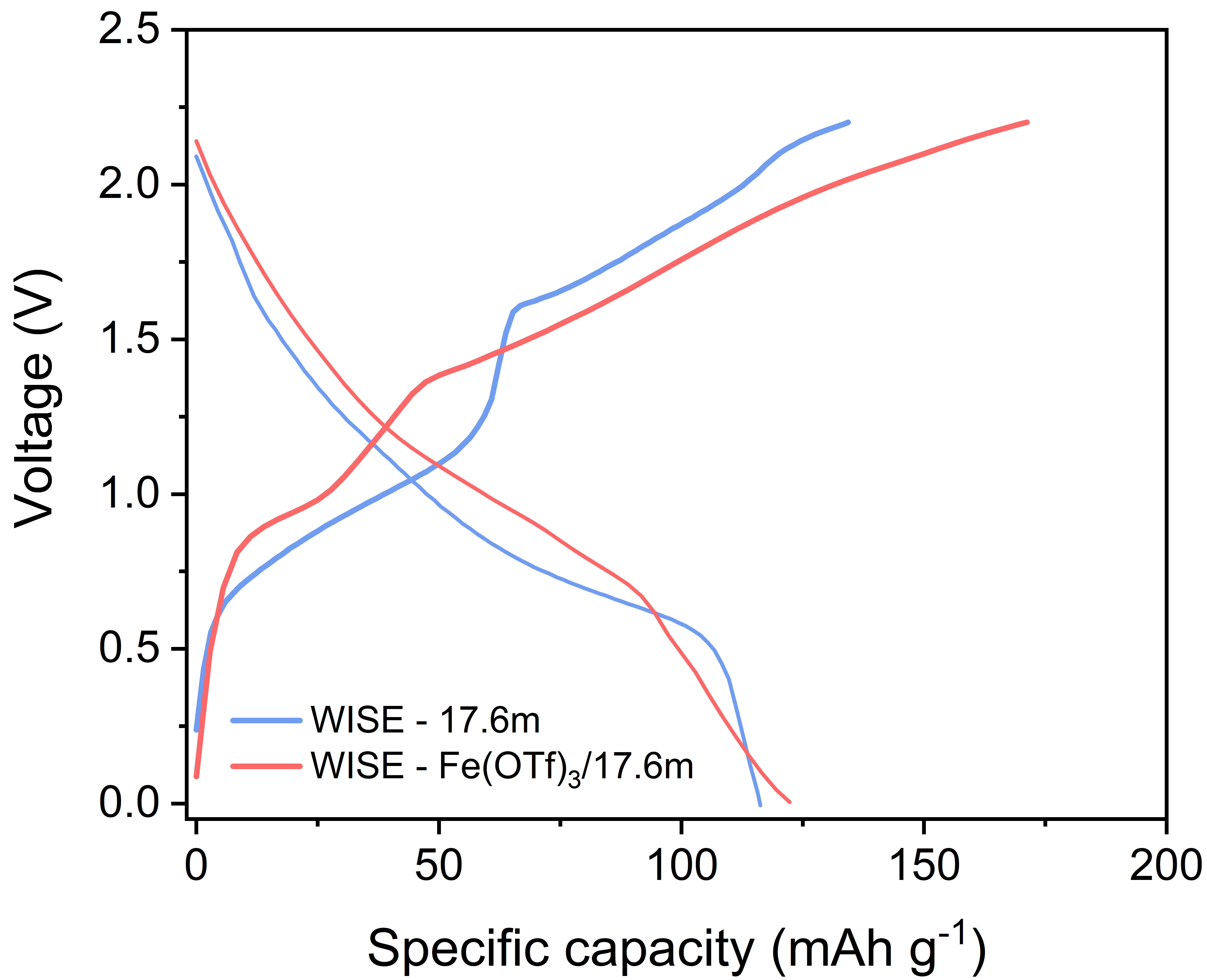


**Fig. S17** Initial charging and discharging curves of Mn-HCF electrodes in WISE-17.6m and WISE-Fe(OTf)_3_/17.6m at 1A g^-1^, respectively.


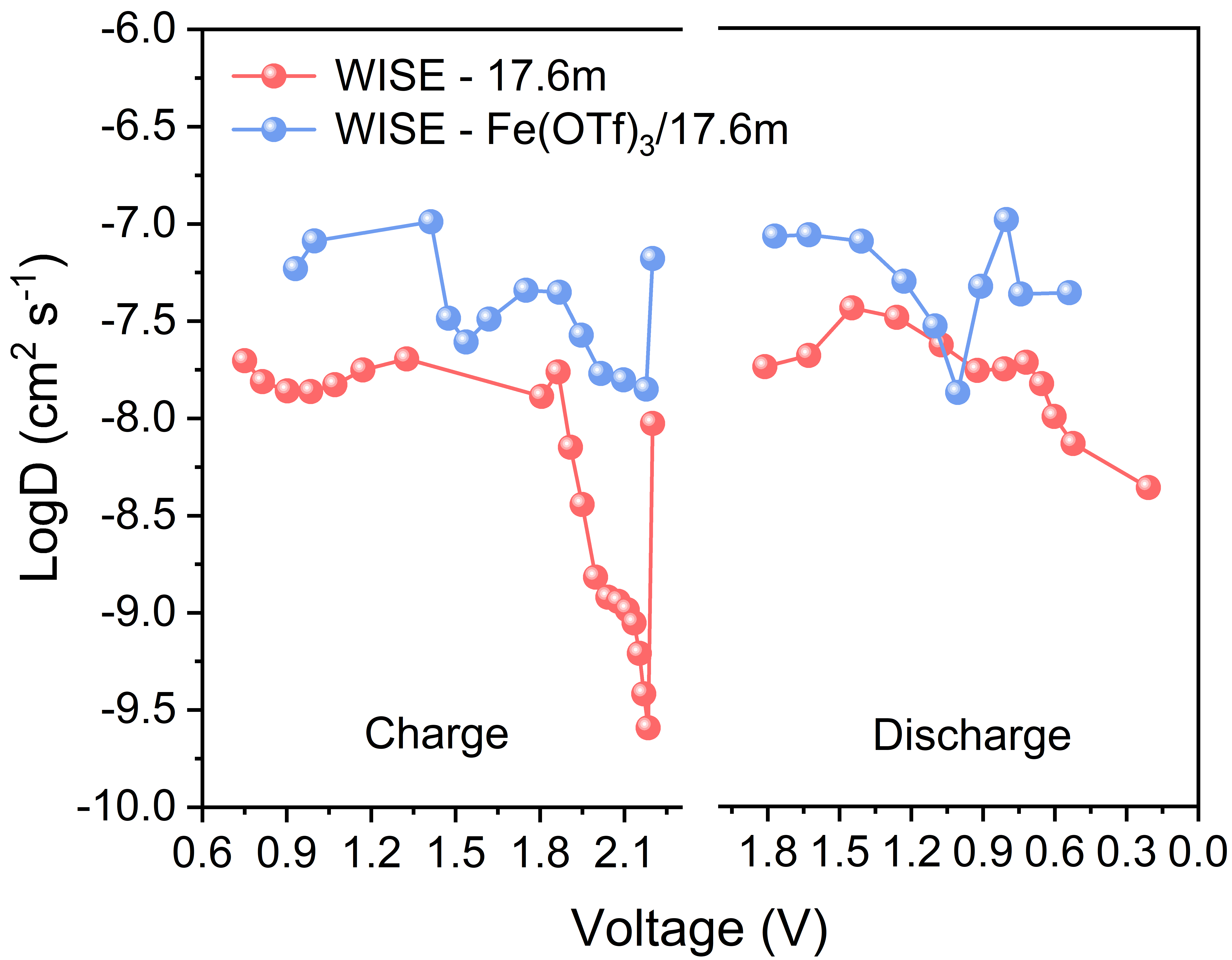


**Fig.S18** Calculated Na^+^ diffusion coefficients from the GITT test for Mn-HCF electrode in WISE-17.6m and WISE-Fe(OTf)_3_/17.6m, respectively.


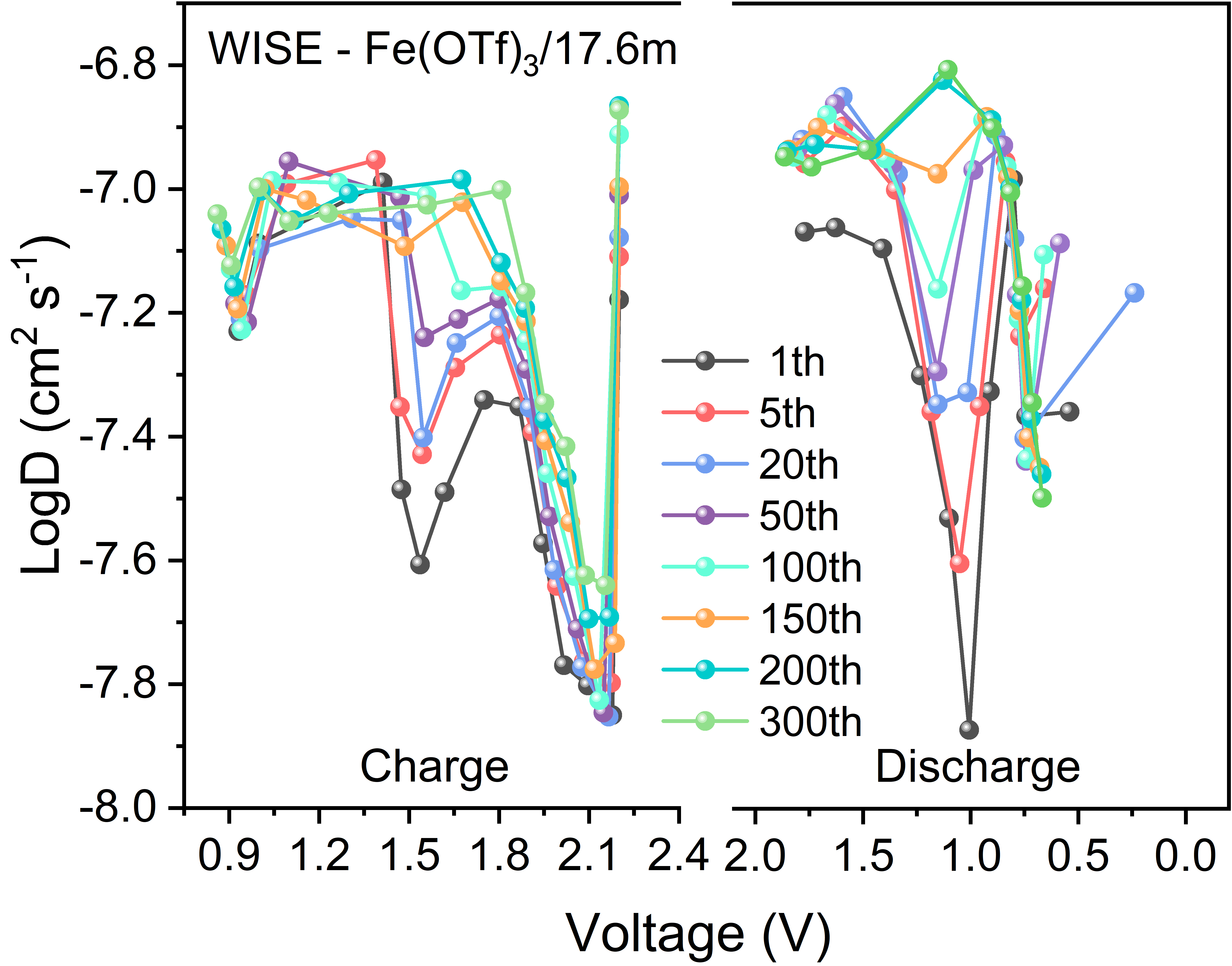


**Fig.S19** Calculated Na^+^ diffusion coefficients from the GITT test for Mn-HCF electrode in WISE-Fe(OTf)_3_/17.6m at different cycle numbers.


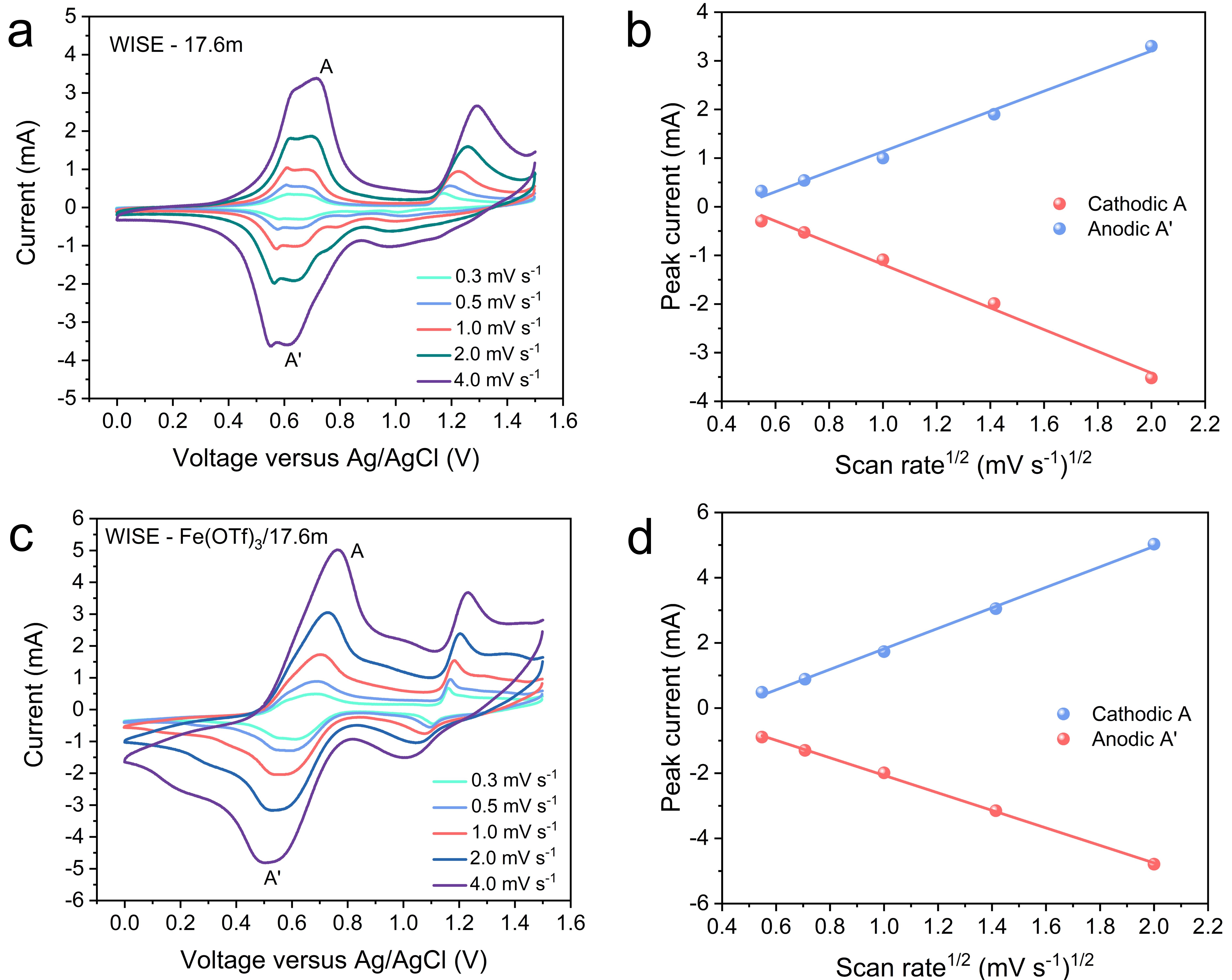


**Fig. S20** CV curves of the Mn-HCF electrodes in the WISE-17.6m (**a)** and WISE-Fe(OTf)₃/17.6m (**c)** electrolytes at various scan rates. Plot of the peak current (*ip*) of Mn-HCF electrodes in the WISE-17.6m (**b)** and WISE-Fe(OTf)₃/17.6m (**d)** electrolytes as a function of the square root of scan rates (*v^1/2^*). The apparent diffusion coefficients of the charge carriers could be calculated to be 4.1×10^-9^ cm^2^ s^-1^ (cathodic peak), and 4.78×10^-9^ cm^2^ s^-1^ (anodic peak B) in the WISE-17.6m electrolytes, 9.5×10^-9^ cm^2^ s^-1^ (cathodic peak) and 5.4×10^-9^ cm^2^ s^-1^ (anodic peak) in the WISE-Fe(OTf)₃/17.6m electrolytes.


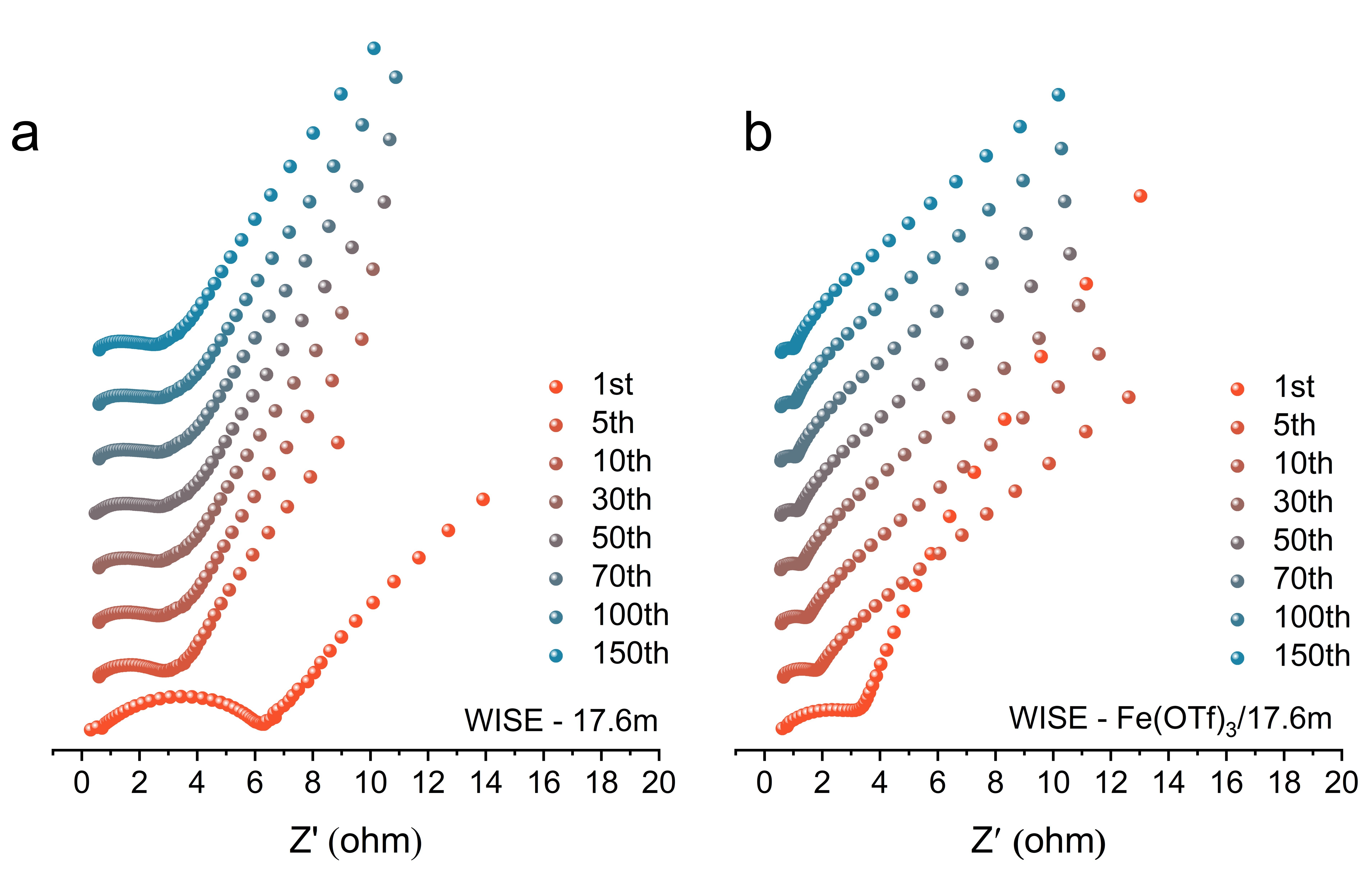


**Fig. S21** EIS spectra of Mn-HCF electrode at different cycle number in (**a**) WISE-17.6m and (**b**) WISE-Fe(OTf)_3_/17.6m, respectively.


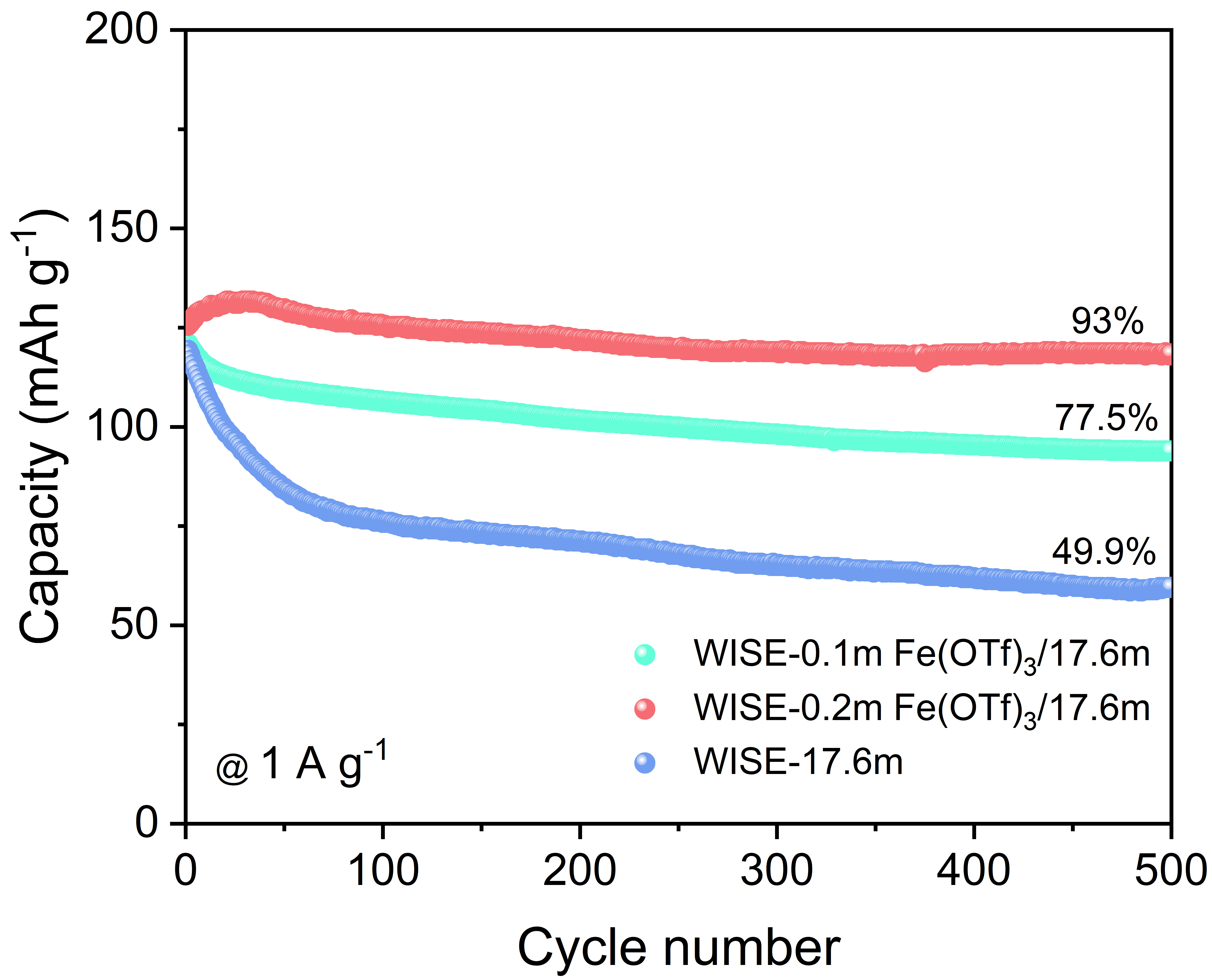


**Fig. S22** Electrochemical performance of the Mn-HCF||PTCDI full cell in WISE-17.6m, WISE-0.1m Fe(OTf)₃/17.6m and WISE-0.2m Fe(OTf)₃/17.6m electrolytes.


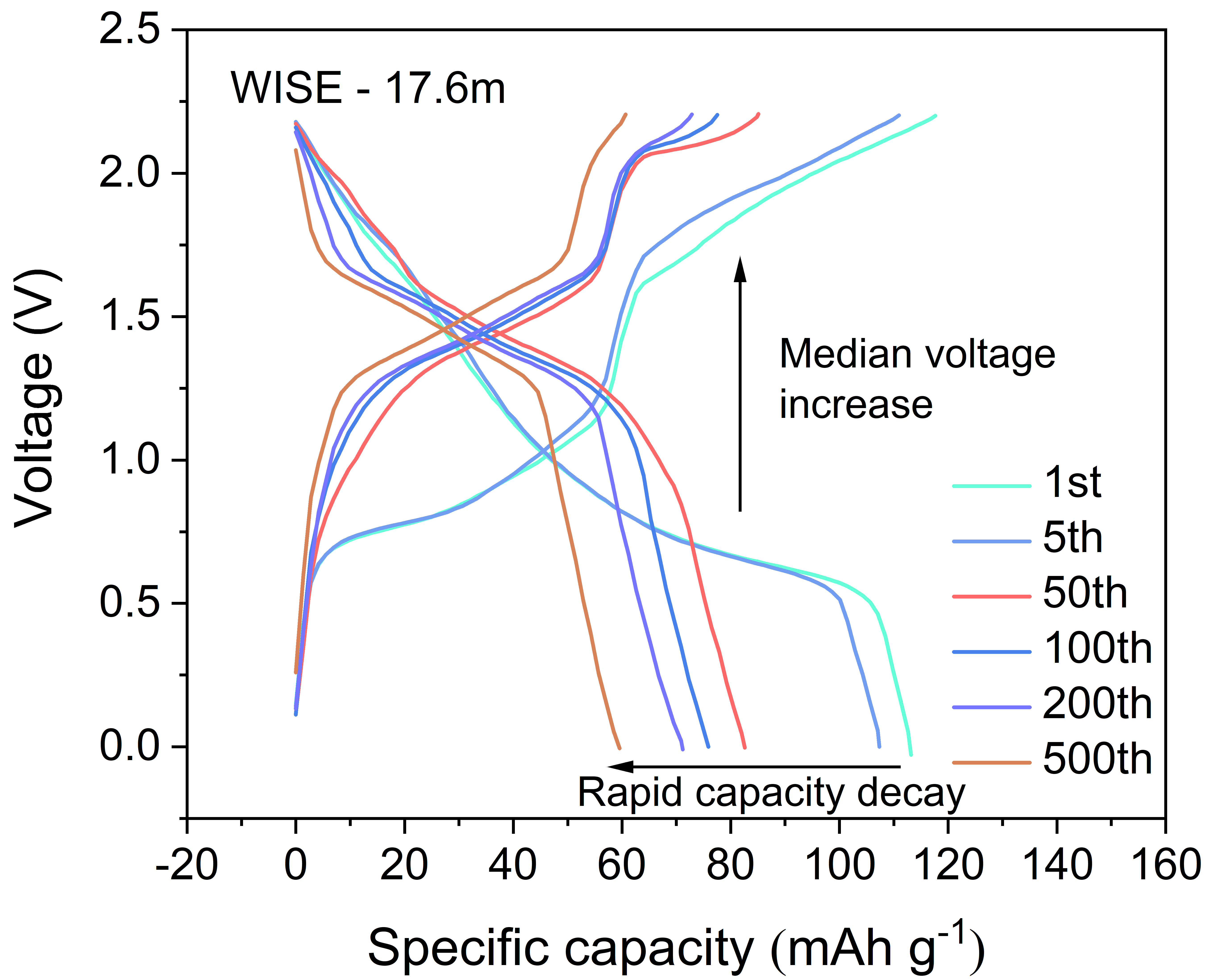


**Fig. S23** Charge-discharge curves of Mn-HCF electrode in WISE- 17.6m at different cycle numbers.


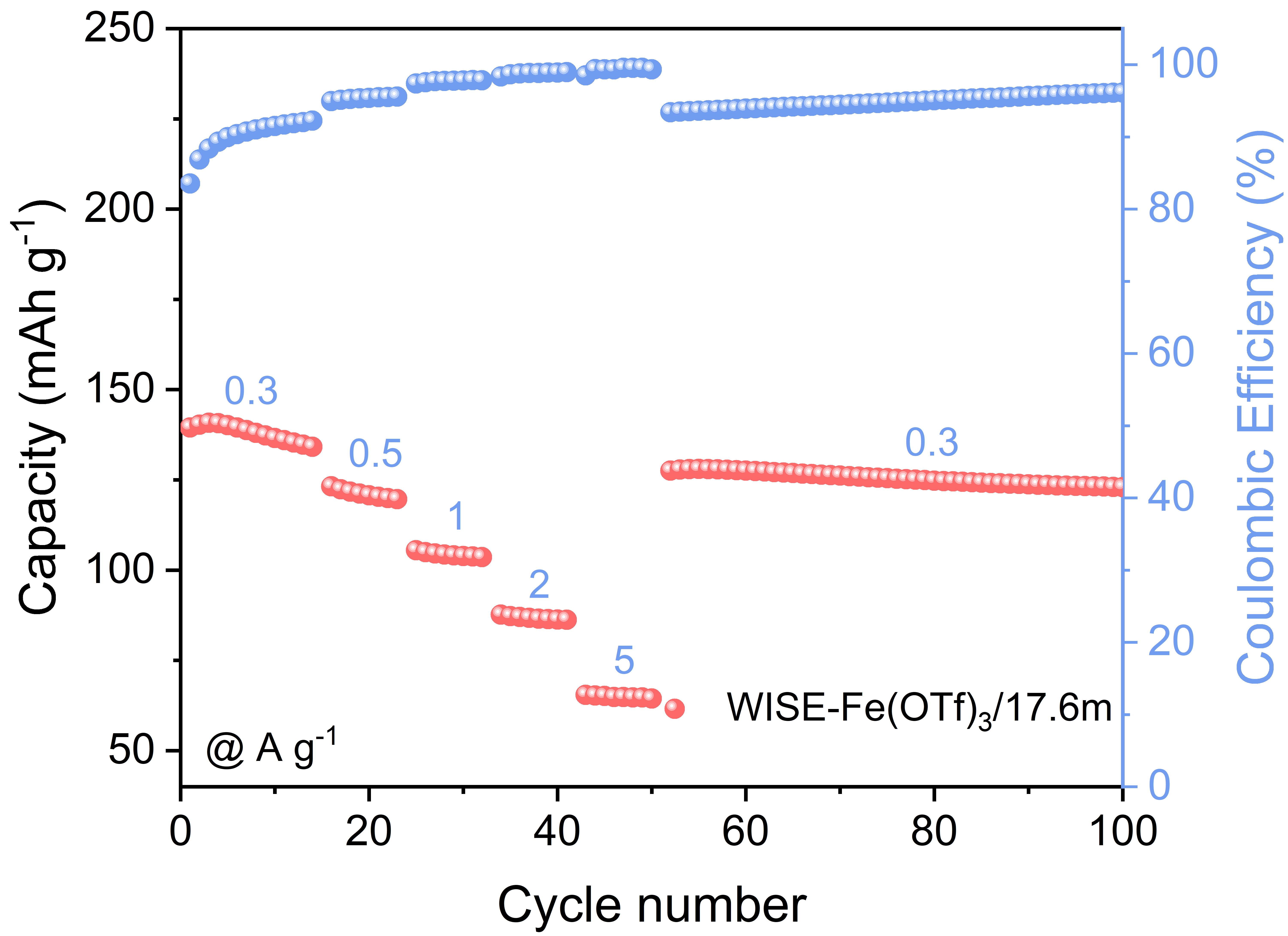


**Fig. S24** Electrochemical performance of the Mn-HCF||PTCDI full cell with WISE-Fe(OTf)_3_/17.6m. The electrode active materials mass loading is 12 mg cm^-^².


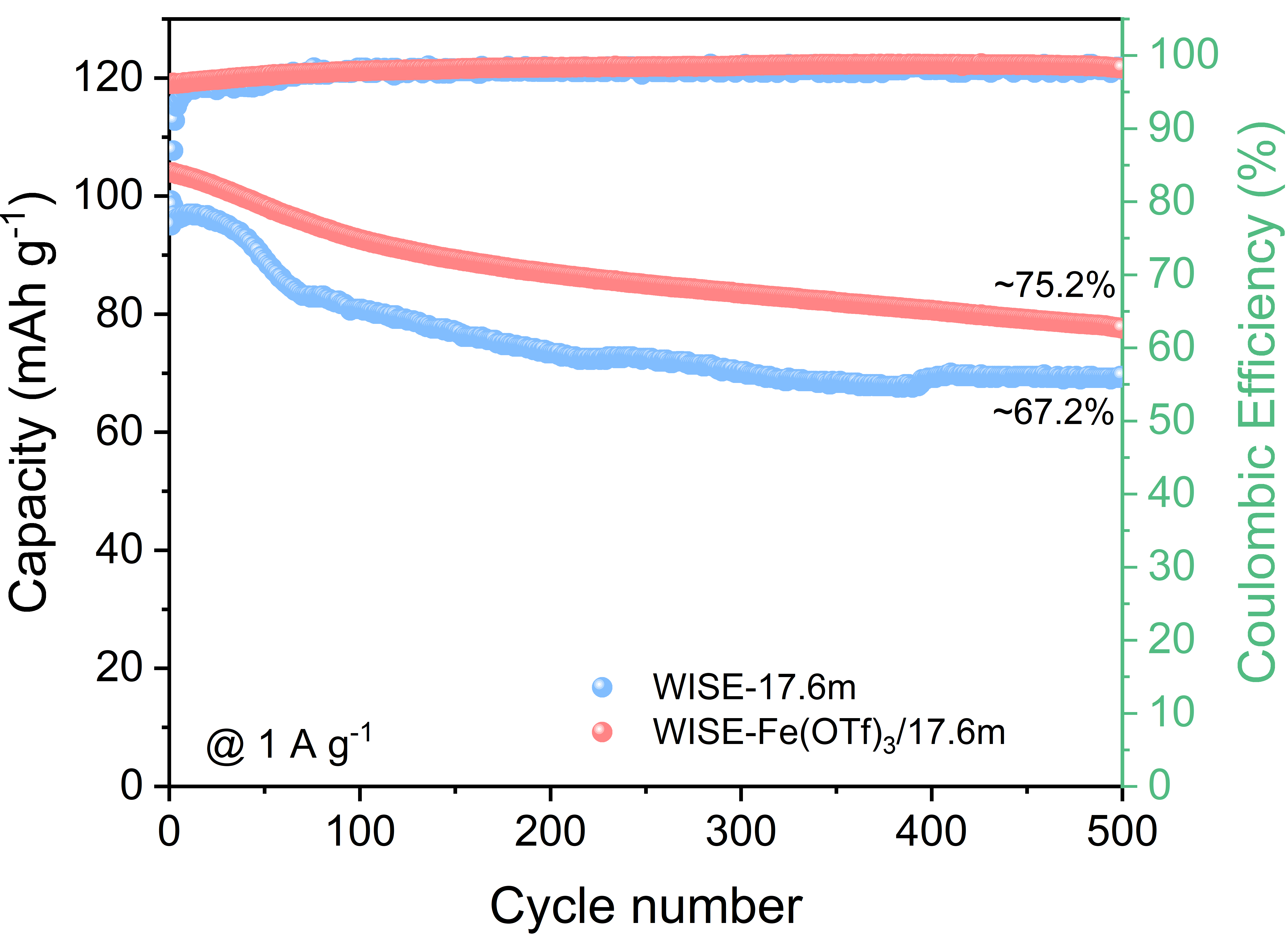


**Fig. S25** Electrochemical performance of the Fe-HCF||PTCDI full cell with WISE-17.6m and WISE-Fe(OTf)_3_/17.6m electrolytes, respectively.


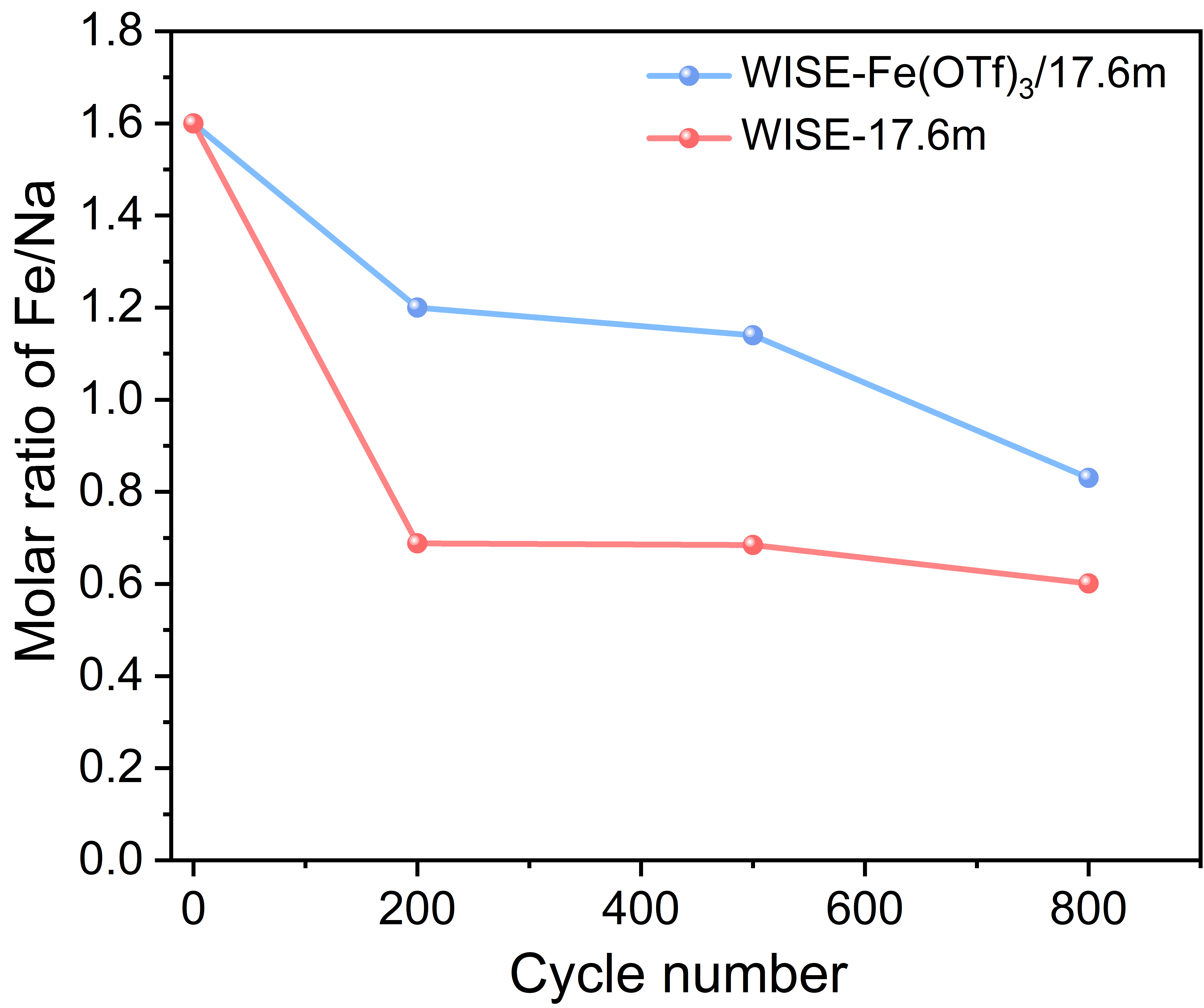


**Fig. S26** Ratio of Fe to Na in the electrode at different cycle numbers.


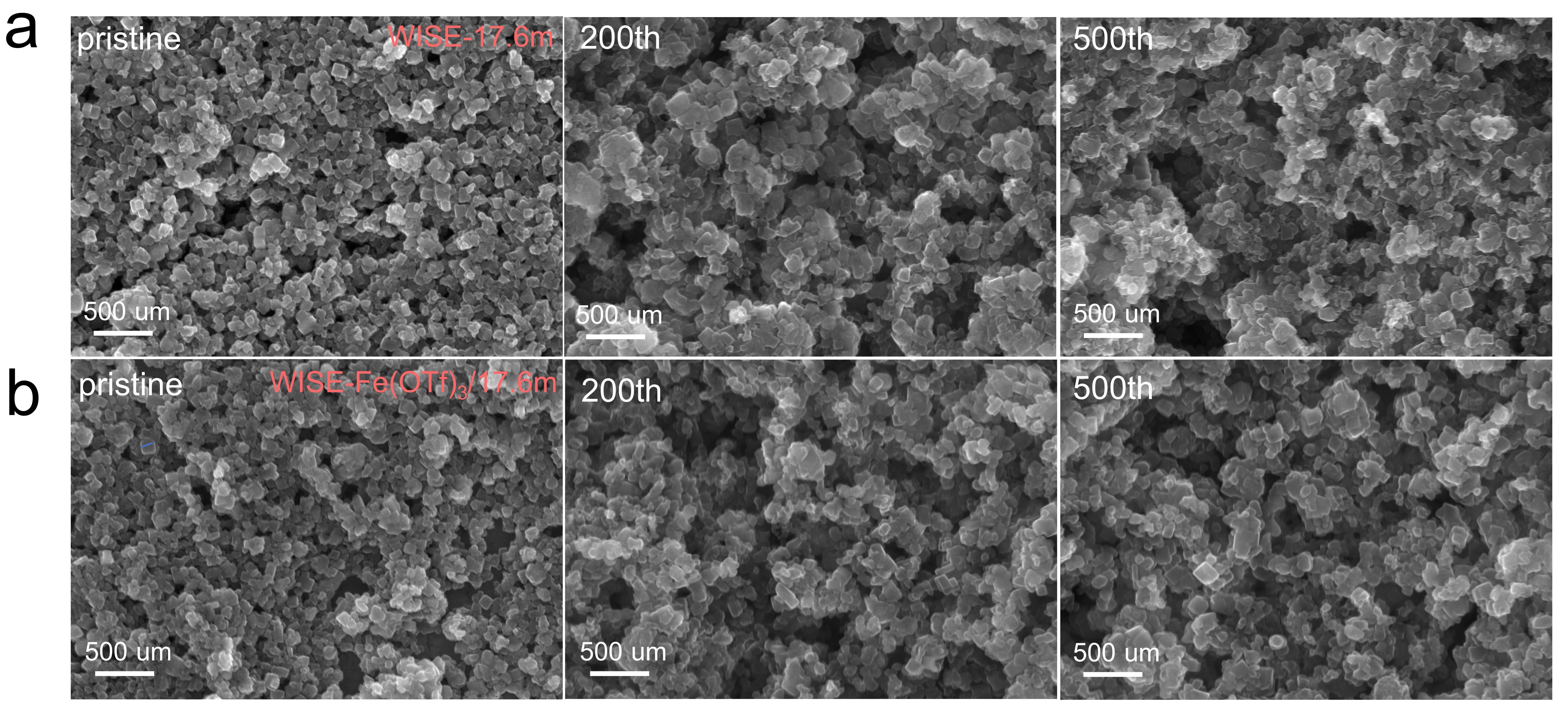


**Fig. S27** SEM images of Fe-HCF electrode after cycling in (**a**) WISE-17.6m and (**b**) WISE-Fe(OTf)_3_/17.6m at different cycle numbers, respectively.

**Table S1** Detailed structural information on the cubic Mn-HCF powder after Rietveld refinement

| Space group: *Fm-3m*; a=b=c=10.50739 Å | | | | | |
| --- | --- | --- | --- | --- | --- |
| Atom | x | y | z | Occupation | Uiso |
| Mn1 | 0.5 | 0 | 0 | 1 | 0.021 |
| Fe1 | 0 | 0 | 0 | 0.699 | 0.020 |
| Na1 | 0.2194 | 0.22 | 0.22 | 0.153 | 0.037 |
| C1 | 0.1753 | 0 | 0 | 0.769 | 0.048 |
| N1 | 0.2911 | 0 | 0 | 0.769 | 0.048 |
| O1 | 0.2911 | 0 | 0 | 0.175 | 0.048 |
| O1 | 0.0463 | 0.25 | 0.25 | 0.287 | 0.117 |

**Table S2** The ICP-AES analysis for Mn-HCF and Fe-HCF (unit: mg/g).

| Sample | Na | Fe | Mn |
| --- | --- | --- | --- |
| Mn-HCF | 83 | 137 | 176 |
| Fe-HCF | 89.2 | 343.9 | - |

**Table S3** Analyzed results of the elemental content of the samples (unit: %)

| Sample | C | H | N |
| --- | --- | --- | --- |
| Mn-HCF | 17.68 | 2.46 | 20.62 |
| Fe-HCF | 17.89 | 1.99 | 20.32 |

**Table S4** Detailed structural information on the cubic Fe-HCF powder after Rietveld refinement

| Space group: *Fm-3m;* a=b=c=10.26274 Å | | | | | |
| --- | --- | --- | --- | --- | --- |
| Atom | x | y | z | Occupation | Uiso |
| Fe1 | 0 | 0 | 0 | 1 | 0.085 |
| Fe2 | 0.5 | 0 | 0 | 0.699 | 0.040 |
| Na1 | 0.21940 | 0.22 | 0.22 | 0.153 | 0.067 |
| C1 | 0.16190 | 0 | 0 | 0.769 | 0.036 |
| N1 | 0.27898 | 0 | 0 | 0.769 | 0.107 |
| O1 | 0.27898 | 0 | 0 | 0.176 | 0. 107 |
| O1 | 0.06427 | 0.25 | 0.25 | 0.287 | 0.145 |

**Table S5** Comparison of lifespan of this work with reported aqueous sodium-ion full batteries.

| **Cathode \|\| Anode** | **Capacity**  **(mAh/g)** | **Cycling** | **Current density (A/g)** | **Refs.** |
| --- | --- | --- | --- | --- |
| NaFeMnF \|\| PTCDI | 157 | 15000 cycles, 73.4% | 2 | [S1] |
| MnHCF-Ni/C \|\| NTP | 116 | 13000 cycles, 74.3% | 1.18 | [S2] |
| FeHCF-NCO \|\| NTP | 144 | 15000 cycles, 85% | 1.7 | [S3] |
| HE-PBA \|\| NTP | 118.6 | 1800 cycles, 81% | 0.17 | [S4] |
| MnHCF \|\| NTOP | 140 | 800 cycles, 76% | 0.14 | [S5] |
| Na_4_Fe(CN)_6_ \|\| NTP | 113 | 1000 cycles, 84.7% | 0.09 | [S6] |
| MnHCF \|\| NTP@C | 75 | 5000 cycles, 83% | 1.7 | [S7] |
| PW \|\| NTP | 122 | 10000 cycles, 70% | 1.4 | [S8] |
| NaCoPB \|\| PTCDI | - | 1000 cycles, 76% | 1.7 | [S9] |
| NaFeMnPB \|\| NTP | - | 5000 cycles, 70% | 1.36 | [S10] |
| MnCo-PBA \|\| NTP@C | 119.94 | 5000 cycles, 85.82% | 0.2 | [S11] |
| KMnHCF \|\| NTP | 108 | 10000 cycles, 80% | 0.2 | [S12] |
| NaMnPB \|\| NTP | 118 | 1000 cycles, 74.5% | 0.5 | [S13] |
| NMO \|\| NTP | 75.16 | 3500 cycles, 85% | 0.36 | [S14] |
| NMF \|\| NTP | 105 | 4000 cycles, 80% | 1.5 | [S15] |
| **MnHCF \|\| PTCDI** | **157.5** | **20000 cycles, 80%** | **2** | **This work** |

Supplementary References

1. Z. Liang, F. Tian, G. Yang, C. Wang, Enabling long-cycling aqueous sodium-ion batteries via Mn dissolution inhibition using sodium ferrocyanide electrolyte additive. Nat. Commun. **14**(1), 3591 (2023). <https://doi.org/10.1038/s41467-023-39385-6>.
2. H. Wu, J. Hao, Y. Jiang, Y. Jiao, J. Liu et al., Alkaline-based aqueous sodium-ion batteries for large-scale energy storage. Nat. Commun. **15**(1), 575 (2024). <https://doi.org/10.1038/s41467-024-44855-6>.
3. C. Xu, Y. Liu, S. Han, Z. Chen, Y. Ma et al., Rational design of aqueous Na ion batteries toward high energy density and long cycle life. J. Am. Chem. Soc. **147**(8), 7039-7049 (2025). <https://doi.org/10.1021/jacs.4c18168>.
4. B. Ran, R. Cheng, Y. Zhong, X. Zhang, T. Zhao et al., High entropy activated and stabilized nickel-based prussian blue analogue for high-performance aqueous sodium-ion batteries. Energy Storage Mater. **71** 103583 (2024). <https://doi.org/https://doi.org/10.1016/j.ensm.2024.103583>.
5. L. Jiang, L. Liu, J. Yue, Q. Zhang, A. Zhou et al., High-voltage aqueous Na-ion battery enabled by inert-cation-assisted water-in-salt electrolyte. Adv. Mater. **32**(2), 1904427 (2020). <https://doi.org/https://doi.org/10.1002/adma.201904427>.
6. W. Zhou, Y. Zheng, M. Zartashia, Y. Shan, H. Noor et al., Aqueous dual-electrolyte full-cell system for improving energy density of sodium-ion batteries. ACS Appl. Mater. Interfaces **14**(30), 34835-34843 (2022). <https://doi.org/10.1021/acsami.2c06304>.
7. X. Zhao, Z. Xing, C. Huang, Investigation of high-entropy prussian blue analog as cathode material for aqueous sodium-ion batteries. J. Mater. Chem. A **11**(42), 22835-22844 (2023). <https://doi.org/10.1039/D3TA04349E>.
8. D. Peng, R. Sun, J. Han, T. Zhao, R. Tian et al., A low-concentrated electrolyte with a 3.5 V electrochemical stability window, made by restructuring the H-bond network, for high-energy and long-life aqueous sodium-ion batteries. ACS Energy Lett. **9**(12), 6215-6224 (2024). <https://doi.org/10.1021/acsenergylett.4c02901>.
9. L. Jiang, Y. Hu, F. Ai, Z. Liang, Y. Lu, Rational design of anti-freezing electrolyte concentrations via freeze concentration process. Energy Environ. Sci. **17**(8), 2815-2824 (2024). <https://doi.org/10.1039/D4EE00859F>.
10. L. Jiang, S. Han, Y. Hu, Y. Yang, Y. Lu et al., Rational design of anti-freezing electrolytes for extremely low-temperature aqueous batteries. Nat. Energy **9**(7), 839-848 (2024). <https://doi.org/10.1038/s41560-024-01527-5>.
11. J. Chen, Y. Chen, F. Ning, W. Shi, Y. Yang, An induced recrystallization self-healing separator for stabilizing ultra-long cycles of aqueous sodium ion batteries. Energy Storage Mater. **79** 104318 (2025). <https://doi.org/https://doi.org/10.1016/j.ensm.2025.104318>.
12. J. Liu, C. Yang, B. Wen, B. Li, Y. Liu, Ultra-long cycle of prussian blue analogs achieved by equilibrium electrolyte for aqueous sodium-ion batteries. Small **19**(46), 2303896 (2023). <https://doi.org/https://doi.org/10.1002/smll.202303896>.
13. T. Liu, H. Wu, X. Du, J. Wang, Z. Chen et al., Water-locked eutectic electrolyte enables long-cycling aqueous sodium-ion batteries. ACS Appl. Mater. Interfaces **14**(29), 33041-33051 (2022). <https://doi.org/10.1021/acsami.2c04893>.
14. Z. Hou, X. Zhang, J. Chen, Y. Qian, L. Chen et al., Towards high-performance aqueous sodium ion batteries: Constructing hollow NaTi_2_(PO_4_)_3_@C nanocube anode with Zn metal-induced pre-sodiation and deep eutectic electrolyte. Adv. Energy Mater. **12**(14), 2104053 (2022). <https://doi.org/https://doi.org/10.1002/aenm.202104053>.
15. Z. Hou, W. Mao, Z. Zhang, J. Chen, H. Ao et al., Bipolar electrode architecture enables high-energy aqueous rechargeable sodium ion battery. Nano Res. **15**(6), 5072-5080 (2022). <https://doi.org/10.1007/s12274-022-4113-0>.
